# A new pseudosuchian archosaur, *Mambawakale ruhuhu* gen. et sp. nov., from the Middle Triassic Manda Beds of Tanzania

**DOI:** 10.1098/rsos.211622

**Published:** 2022-02-09

**Authors:** Richard J. Butler, Vincent Fernandez, Sterling J. Nesbitt, João Vasco Leite, David J. Gower

**Affiliations:** ^1^ School of Geography, Earth and Environmental Sciences, University of Birmingham, Birmingham, UK; ^2^ Department of Geosciences, Virginia Tech, Blacksburg, VA, USA; ^3^ Imaging and Analysis Centre, Natural History Museum, London, UK; ^4^ Department of Earth Sciences, Natural History Museum, London, UK; ^5^ Department of Life Sciences, Natural History Museum, London, UK

**Keywords:** Archosauria, Triassic, Tanzania, phylogeny, Pseudosuchia

## Abstract

The Manda Beds of southwest Tanzania have yielded key insights into the early evolutionary radiation of archosaurian reptiles. Many key archosaur specimens were collected from the Manda Beds in the 1930s and 1960s, but until recently, few of these had been formally published. Here, we describe an archosaur specimen collected in 1963 which has previously been referred to informally as *Pallisteria angustimentum*. We recognize this specimen as the type of a new taxon, *Mambawakale ruhuhu* gen. et sp. nov. The holotype and only known specimen of *M. ruhuhu* comprises a partial skull of large size (greater than 75 cm inferred length), lower jaws and fragments of the postcranium, including three anterior cervical vertebrae and a nearly complete left manus. *Mambawakale ruhuhu* is characterized by several cranial autapomorphies that allow it to be distinguished with confidence from all other Manda Beds archosaurs, with the possible exception of *Stagonosuchus nyassicus* for which comparisons are highly constrained due to very limited overlapping material. Phylogenetic analysis suggests that *M. ruhuhu* is an early diverging pseudosuchian, but more precise resolution is hampered by missing data. *Mambawakale ruhuhu* is one of the largest known pseudosuchians recovered to date from the Middle Triassic.

## 1. Introduction

One of the key transitions in terrestrial vertebrate evolution occurred during the Triassic, when the dominant synapsids of the preceding Permian Period were replaced by the earliest members of a new clade, Archosauria [1,2], as well as their stem lineage, non-archosaurian archosauromorphs [3]. Archosaurs, which include living birds and crocodilians as well as the extinct pterosaurs and non-avian dinosaurs, originated by the end of the Early Triassic [4,5], but their major initial radiation occurred during the Middle Triassic [2,5,6]. Abundant fossil material documenting the early stages of the archosaur radiation has been collected from the Manda Beds of the Ruhuhu Basin, southwest Tanzania, which have been generally considered to date to the Anisian stage of the Middle Triassic [7–21], although an alternative hypothesis places the Manda Beds as young as the Carnian stage of the Late Triassic [22,23].

The first collections of archosaur material from the Manda Beds to be reported in the scientific literature were made in the 1930s by Gordon Murray Stockley of the Tanganyika Geological Survey and Francis Rex Parrington of the University of Cambridge, while other tetrapod material was collected in the same decade by the Austrian geologist Ernst Nowack [7–9,24]. At this point, Tanzania was a territory within the British Empire known as Tanganyika, and these discoveries of fossil vertebrates in the Ruhuhu Basin were linked to the extraction of natural resources [7]. These colonial era collections of material from the Manda Beds were reposited in museums in South Africa (Iziko South African Museum, Cape Town), the UK (Natural History Museum, London; University Museum of Zoology, Cambridge) and Germany (Institut für Geologie und Paläontologie, Tübingen; Bayerische Staatssammlung für Paläontologie und Geologie, Munich).

Much of the archosaur material collected in the 1930s by Parrington was brought to the UK and described by Alan Charig in the 1950s in his unpublished PhD dissertation supervised by Parrington at the University of Cambridge [25]. Although Charig went on to a long career at the Natural History Museum in London, he did not publish his work on the archosaurs from the Manda Beds in his lifetime [26]. Only after Charig's death were many of Parrington's key specimens formally published [11,13,15,17].

**Figure 1.**
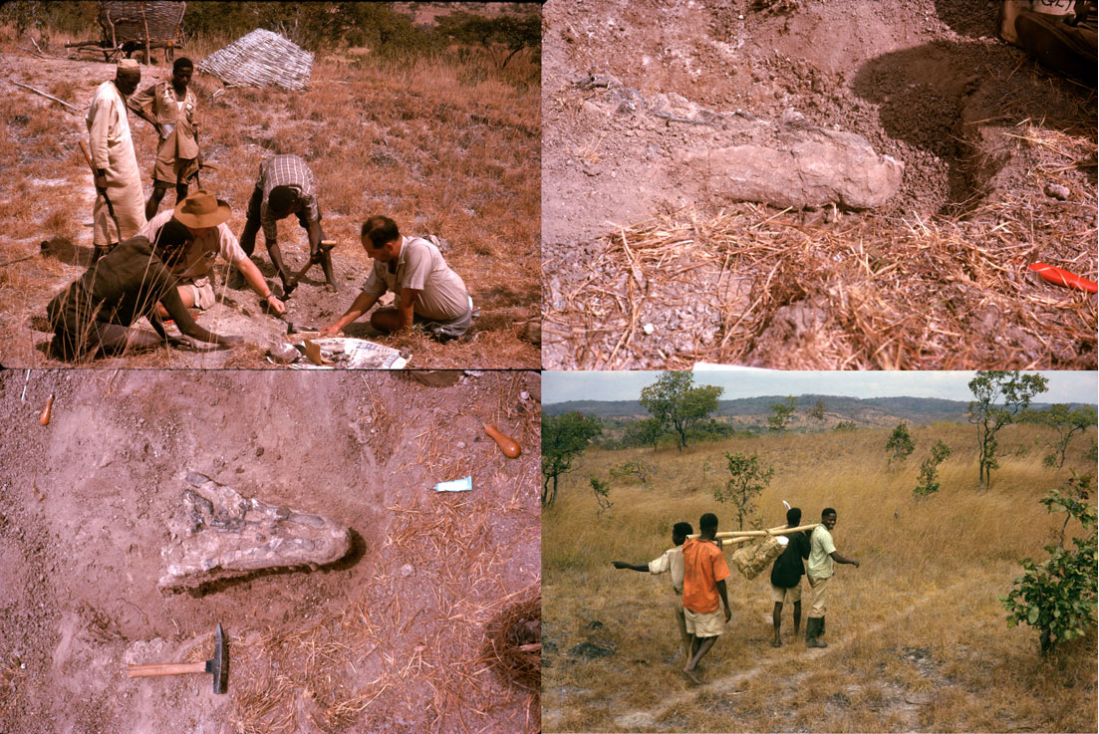
Photographs showing the collection of NHMUK R36620, holotype of *Mambawakale ruhuhu*, in 1963. Alan Charig is sat in the bottom right of the frame in the top left image, and is accompanied by Alfred ‘Fuzz’ Crompton. The top left and bottom right images also show Tanzanians (names unfortunately not recorded in archival material) who were employed by the 1963 expedition team and were critical to its success, discovering many of the fossil sites, constructing roads and carrying excavated fossils out of the field. Photographs courtesy of Barry Cox and Steve Tolan. Original slides of these photographs are archived at NHMUK.

In 1963, 2 years after Tanganyika gained independence and 1 year before it would be renamed as Tanzania, Charig participated in the British Museum (Natural History) (= today's Natural History Museum, London) and University of London joint expedition to modern-day Tanzania and Zambia [20,27–29]. This expedition also involved researchers from the South African Museum (Cape Town), the Uganda Museum (Kampala) and the University of Edinburgh, and was heavily reliant on support from Tanzanians and Zambians, who found many of the localities from which fossils were collected, found fossils at those localities, and who were also employed to build roads for the passage of expedition vehicles and to transport fossils from the field [27–29]. Unfortunately, these Tanzanians and Zambians are unnamed in published reports and field notes, although photographs show some of them contributing to the field work (figure 1). Fossils collected on this 1963 expedition were returned to the UK, where they are held in the collections of the Natural History Museum.

The 1963 expedition collected significant new vertebrate material from the Manda Beds, including what would become the holotype of the pseudosuchian archosaur *Hypselorhachis mirabilis* [10]. Attridge *et al*. [27] provided a report on the discoveries made during this expedition and mentioned an ‘incomplete skull and jaws 2 ft. long superficially similar to those of a large modern crocodile, with 4½-in. teeth’ ([27]: p. 447, fig. 4). This specimen (NHMUK R36620) was subsequently referred to as *Pallisteria angustimentum* Charig, 1967 in the ‘Reptilia’ chapter of *The Fossil Record* (Charig in [30]: p. 708). It was placed within the family ‘Pallisteriidae Charig, 1967’, and suggested to be ‘possibly close to line of ancestry of crocodilians' (ibid.). No details, figures or specimen numbers were provided. ‘Charig, 1967’ refers to a manuscript listed as ‘in press’ at the journal *Palaeontology*; however, this manuscript was never published, nor has such a manuscript been identified in the archival material of Charig held by the Natural History Museum (R.J.B. personal observation; A. Milner 2019, personal communication). As such, *Pallisteria* and *Pallisteria angustimentum* are nomina nuda. *Pallisteria* has subsequently been mentioned in passing in the literature several times (e.g. [20,31–33]), but has never received an anatomical description, and is the last significant archosaur material preliminarily reported by Charig [25] to remain unpublished, following the publication of *Hypselorhachis*, *Nyasasaurus*, *Teleocrater* and *Mandasuchus* [10,11,13,15].

The aim of this paper is to provide the first description of this specimen, previously referred to as *Pallisteria angustimentum*. We formally diagnose it as a new taxon, *Mambawakale ruhuhu* gen. et sp. nov., and infer its phylogenetic position.

*Institutional abbreviations*.

CPEZ, Paleontology Collection of the Museu Paleontológico e Arqueológico Walter Ilha, São Pedro do Sul, Brazil; NHMUK, Natural History Museum, London, UK; UFRGS PV, Paleovertebrate Collection of the Laboratório de Paleovertebrados of the Universidade Federal do Rio Grande do Sul, Porto Alegre, Brazil.

## 2. Material and methods

The skull and mandible of NHMUK R36620 were characterized by V.F. with X-ray micro-computed tomography (CT), using a Nikon HMX ST 225 (Nikon Metrology NV, Leuven, Belgium) at NHMUK. Because the specimens were larger than the maximum field of view available, several acquisitions were made separately and merged later in the post-processing. In total, the skull required three separate acquisitions, and each hemimandible required four acquisitions. Two of the three acquisitions for the skull were done with the snout pointing down, moving the specimen along the vertical axis of the motorized sample manipulator. The specimen was then flipped, snout pointing up, to image the posterior portion of the skull. All three skull acquisitions had similar parameters: tungsten static reflection target; voltage of 225 kV and current of 600 µA; filtration with 2 mm of silver; 3142 projections recorded over a rotation of 360°; four frames averaged per projection, 1 s exposure time each; source to object distance of 744.1 mm and source to detector distance of 1173.8 mm, achieving an isotropic voxel size of 126.8 µm in the reconstructed data. Setting each hemimandible vertically on the sample stage, two acquisitions of the posterior parts were performed first, before flipping the fossils upside down and performing two additional acquisitions for the anterior part. CT settings comprised: tungsten rotating reflection target; voltage of 180 kV and current of 367 µA; filtration with 1 mm of tin; 1470 projections recorded over a rotation of 360°; 250 ms exposure time per projection; two frames averaging; source to object distance of 680.9 mm and a source to detector distance of 1128.1 mm, 2 × 2 binning of the detector, achieving an isotropic voxel size of 181.1 µm in the reconstructed data.

The CT reconstruction was performed using CT pro 3D 6.0 (Nikon Metrology NV, Leuven, Belgium). All datasets were first reconstructed as 32-bit volumes. The merging of the datasets (three datasets for the skull and four datasets for each hemimandible) was done in two stages. First, we merged pairs of datasets when the specimens were moved along the vertical axis using motors of the sample stage. Second, we merged the datasets representing the anterior and posterior parts of each fossil, obtained by turning the specimens upside-down. The first merging process used an in-house script written in Octave 4.4.1. The workflow of the merging script consisted of first an estimation of the overlap between the two datasets based on motor positions, and then a fine tuning based on best matching of pairwise images. The first estimation provided a gross estimate of the number of slices the datasets have in common; for the ‘top’ dataset, a ‘reference’ image was defined, located in the middle of the estimated overlapping portion; for the ‘bottom’ dataset, a range of 30 ‘target’ images was selected, surrounding the estimated middle value of the overlap. After applying a high-pass filter on images, each image from the ‘target’ range was sequentially subtracted to the ‘reference’ image, each time measuring the resulting standard deviation. The pair of images providing the smallest standard deviation values indicated the best fit for overlap between the two datasets. The overlapping part between the two datasets was handled with a weighted average of the analogous images, allowing for a smooth transition from one dataset to the other. The merged dataset was then exported as a stack of 16-bit tiffs, the change in dynamic range being based on maximum and minimum values of the 32-bit histogram determined manually by the user (here done in ImageJ; [34]). For each hemimandible, we ensured that the change in dynamic range was done similarly for the anterior and posterior part. The posterior portion of the skull from the third acquisition was reconstructed directly as a 16-bit raw volume, using the same maximum and minimum values for scaling down the 32-bit data. Because the second merging process involved both translation and rotation (specimen manually turned upside-down), we used the tool ‘Geometric transform/Register Images' in Avizo 2019.1 (Thermo Fisher Scientific, Hillsborough, OR, USA); the registration was undertaken in rigid mode and in three dimensions. After the registration was completed, the datasets were merged with the tool ‘Compute/Volume Operations/Merge’; finally, the merged datasets were exported as stacks of 16-bit tiff files.

The segmentation of the bones of the skull was done using VGStudioMax 3.3 (Volume Graphics, Heidelberg, Germany), mostly using a thresholded two-dimensional brush. Each segmented bone was converted to a three-dimensional mesh model using the ‘grid-based—precise and watertight’ preset selection, with no simplification. Each complete hemimandible were converted to three-dimensional mesh models as well, without segmenting individual bones. The three-dimensional mesh models were then imported into Blender 2.9 (Blender, Amsterdam, The Netherlands) to simplify them and smooth jagged surfaces resulting from the manual segmentation. A combination of two ‘remesh’ modifiers was used: first a ‘smooth’ remesh with an Octree Depth value ranging from 7 to 9 depending on the case; second a ‘voxel’ remesh, adjusting the voxel size to simplify the mesh and limit its final size. Rendering of the bones for figures was also carried out using Blender 2.9.

The surfaces of the bones of the manus of *Mambawakale ruhuhu* were scanned by J.V.L. with a Creaform Go!SCAN20 portable surface scanner at 20 mm resolution. Given the small size of the elements, each was scanned twice—dorsal and ventral surfaces—and then merged to form a complete three-dimensional model. Processing, merging and remeshing was performed in VxElements 7.0.2 (Creaform, Québec, Canada) and Geomagic Wrap 2017.0.2 (3D Systems, Rock Hill, USA). Merged CT data for the skull and hemimandibles, three-dimensional mesh models of individual cranial elements, three-dimensional mesh models of the hemimandibles and three-dimensional mesh models of the manual elements are all available to download at MorphoSource (https://www.morphosource.org/projects/000392213). Photographs of the holotype specimen of *Mambawakale ruhuhu* taken by the NHM Photographic Unit are used in the figures herein, and are available to download from the NHM Data Portal (https://doi.org/10.5519/tb5zl1fq).

We assessed the phylogenetic position of *Mambawakale ruhuhu* using the early archosaur matrix of Nesbitt [6] as a framework, and following the modifications of characters, scores and terminal taxa presented by Nesbitt *et al*. [35]. This modified matrix incorporates the largest published taxon and character sampling for early pseudosuchians, as well as an extensive sample of early avemetatarsalians. No new characters were added for the current analysis. *Mambawakale ruhuhu* was the only new taxon to be added and no other taxon scores were modified. In total, there are 439 characters in the current dataset (electronic supplementary material) of which the multistate characters 32, 52, 121, 137, 139, 156, 168, 188, 223, 247, 258, 269, 271, 291, 297, 314 328, 356, 371, 399 and 413 were ordered because they included morphologically intermediate states.

The primary dataset initially included 101 terminal taxa. Following Nesbitt *et al*. [35], we *a priori* excluded the following terminal taxa: *Lewisuchus admixtus* and *Pseudolagosuchus majori* (combined into *Lewisuchus*/*Pseudolagosuchus* following [6,12], *Prestosuchus loricatus* paralectotype, *Prestosuchus chiniquensis* lectotype, *Prestosuchus chiniquensis* paralectotype, *Prestosuchus chiniquensis* type series, UFRGS PV 156T, UFRGS PV 152T and CPEZ 239b were all combined in the *Prestosuchus chiniquensis* ALL terminal taxon following Desojo *et al*. [36] and *Nundasuchus songeaensis* and *Pagosvenator candelariensis* were excluded based on rationale from Nesbitt *et al*. [35]. This resulted in a primary dataset following taxon deletion with 90 taxa. In a second dataset, we included *Nundasuchus songeaensis* to test its relationships with the other Manda Beds taxa. This dataset therefore had a total of 91 taxa. *Nundasuchus songeaensis* was not included in the first analysis because its phylogenetic position is not stable (see [14]) and it may be distantly related to paracrocodylomorphs [14,37].

The matrices were analysed with equally weighted parsimony using PAUP* v. 4.0 (build 169) [38] using heuristic searches with 1000 random addition replicates. Branch support was assessed using non-parametric bootstrapping (100 pseudoreplicates, TBR branch swapping and 10 random addition sequences), and decay indices (Bremer support values) were calculated manually in PAUP* by accepting trees progressively longer than the maximally parsimonious one identified in the original analysis; consensus trees were produced from the results of each of these analyses. Consistency (CI) and retention (RI) indices were calculated in PAUP*. The non-archosauriform archosauromorph *Mesosuchus browni* was set as the outgroup. Zero-length branches were collapsed if they lacked support under any of the most parsimonious reconstructions. Character optimizations for selected nodes in the first analysis were conducted in PAUP* and are presented as electronic supplementary material.

**Systematic Palaeontology**

Archosauria [39] 1869–1870

Pseudosuchia [40] 1887–1890

*Mambawakale ruhuhu* gen. et sp. nov.

‘an incomplete skull and jaws 2 ft. long…’ [27]: p. 448, fig. 4

*Pallisteria angustimentum* Charig 1967 Charig in [30]: p. 708

*Pallisteria* [31]: p. 7

*Pallisteria* [41]: p. 16

*Pallisteria* [42]: p. 52

*Pallisteria* [43]: p. 620

*Pallisteria angustimentum* [44]: p. 17

*Pallisteria* [32]: appendix

*Pallisteria* [10]: p. 1023

*Pallisteria* [45]: p. 47

*Pallisteria* [26]: fig. 3

*Pallisteria angustimentum* [33]: tab. 1

*Pallisteria angustimentum* Charig 1967 [46]: p. 5

*Pallisteria angustimentum* [14]: p. 1359

*Pallisteria angustimentum* [19]: p. 926

*Pallisteria* [20]: fig. 1M

*Pallisteria angustimentum* [47]: p. 30

*Pallisteria* [21]: pp. 1, 6

*Nomenclatural acts.* This publication and its nomenclatural acts are registered at ZooBank. The publication is registered under LSID urn:lsid:zoobank.org:pub:350E1C4A-148A-46ED-A2BD-CB9102DC4D7E, the new genus *Mambawakale* under LSID urn:lsid:zoobank.org:act:2DA9C12B-C23D-4946-82FA-781830E0EB36, and the specific name *M. ruhuhu* under LSID urn:lsid:zoobank.org:act:5D3965B9-3E7B-4DC3-9296-9E113F0572D9.

*Holotype*. NHMUK R36620, partial skull including premaxillae, maxillae, vomers, palatines, pterygoids, ectopterygoids and fragments of the jugals and basipterygoid, with associated hemimandibles, hyoids and isolated maxillary or dentary teeth. These cranial remains are associated (see below) with an incomplete postcranium, including an atlantal intercentrum, partial axis and partial third cervical vertebra, a mostly complete left manus, and additional poorly preserved fragments.

*Etymology*. *Mambawakale*, from the Kiswahili words *mamba*, meaning crocodile, and *wakale*, meaning ancient. The species name refers to the Ruhuhu Basin from which the type specimen and other taxa from the Manda Beds were collected.

Alan Charig originally proposed naming the genus *Pallisteria* in honour of the geologist John Weaver Pallister OBE (1912–1985). Pallister was a friend of Charig and Commissioner of Mineral Resources of Tanganyika (= Tanzania of today) at the time of the 1963 expedition that collected the type specimen of *Pallisteria* and provided some support to the organization of this expedition although he did not take part in it. Pallister worked as a geologist in Zambia, Malaysia, Papua New Guinea, Australia, India, Uganda and Somalia and lectured at Birmingham University (1946–1950), before becoming Director of the Geological Survey of Tanganyika (1960–1965). Following his return to the UK, he worked for the Institute of Geological Sciences (now the British Geological Survey). Charig also originally formulated the proposed species name *angustimentum*, derived from the Latin *angustus*, meaning ‘narrow’, and *mentum*, meaning ‘chin’. This was presumably a reference to the narrow and tapering anterior end of the lower jaw, resulting from the comparatively elongate mandibular symphysis.

Although we recognize Charig's original intention, we prefer instead to use a name of Kiswahili derivation to formally recognize the substantial and previously unsung contributions of unnamed Tanzanians to the success of the 1963 expedition. Given that the names *Pallisteria* and *P. angustimentum* are nomina nuda and have never been discussed in detail in the literature (with approx. 17 mentions in passing or in faunal lists in more than 50 years) there is no strong argument from historical continuity for retaining the name. Aware that our authorship includes no Tanzanians or Kiswahili speakers, the names *Mambawakale* and *M. ruhuhu* were constructed and chosen in consultation with Tanzanian neoherpetologist John Lyakurwa of the University of Dar es Salaam, Tanzania. An audio file of the pronunciation of the new taxon name (recorded by John Lyakurwa) is available from the NHM Data Portal (https://doi.org/10.5519/tb5zl1fq).

*Locality and stratigraphy*. Field locality U15/1 of Attridge *et al*. [27], Lifua Member of the Manda Beds (Middle Triassic: ?Anisian), Ruhuhu Basin, southwest Tanzania. Cox [48] provided a map showing the localities sampled by Attridge *et al*. [27]. Field notes held at NHMUK indicate that the field team considered the skull of *Mambawakale ruhuhu* to be associated with the left manus, as well as ‘numerous other fragments', and photographs show the collection of the specimen (figure 1). These other fragments were collected on the surface near the specimen but are generally highly weathered and most are not currently identifiable. No other skull elements missing from the holotype were readably identified. A partial silesaurid femur (NHMUK R16303), described by Barrett *et al*. [19], is also known from this site, and was identified among the material thought to be associated with the holotype skull of *M. ruhuhu*. Locality U15 appears to have been a larger locality divided by the field team into several sub-localities, which according to the field notes also yielded anomodont cranial remains (U15/2, U15/3), cynodont fragments (U15/4) and bivalves (referred to *Unio karrooensis* in the field notes; U15/5).

The locality has not been relocated with certainty by more recent visits to the area [20], but given the proximity to the mountains in the pictures taken during the excavation (figure 1), the nature of preservation of the fossil material, and the associated fauna, we infer that *M. ruhuhu* is from the upper part of the Lifua Member of the Manda Beds at a similar level to most vertebrate discoveries from the Manda Beds (see [15]).

*Diagnosis*. Large pseudosuchian (skull length greater than 75 cm) diagnosed by the following unique combination of characters (*indicates autapomorphies): premaxillary teeth 1–3 substantially smaller than premaxillary tooth 4*; strongly transversely thickened posterodorsal process of premaxilla*; interdigitating suture between the premaxilla and the maxilla; external naris very large relative to antorbital fenestra and bordered partly by the maxilla; antorbital fossa weakly defined on the lateral side of the maxilla; elongate dentary symphysis extending posteriorly as far as dentary tooth position 8*. See Discussion for differentiation from other Manda Beds taxa.

*Ontogenetic status*. The age of the holotype cannot be assessed using the criteria of Griffin *et al*. [49] because the elements recovered cannot be confidently assessed for age based on a single individual. Elements suitable for histological analysis are not preserved in the holotype.

## 3. Description

### 3.1. Skull

**Figure 2.**
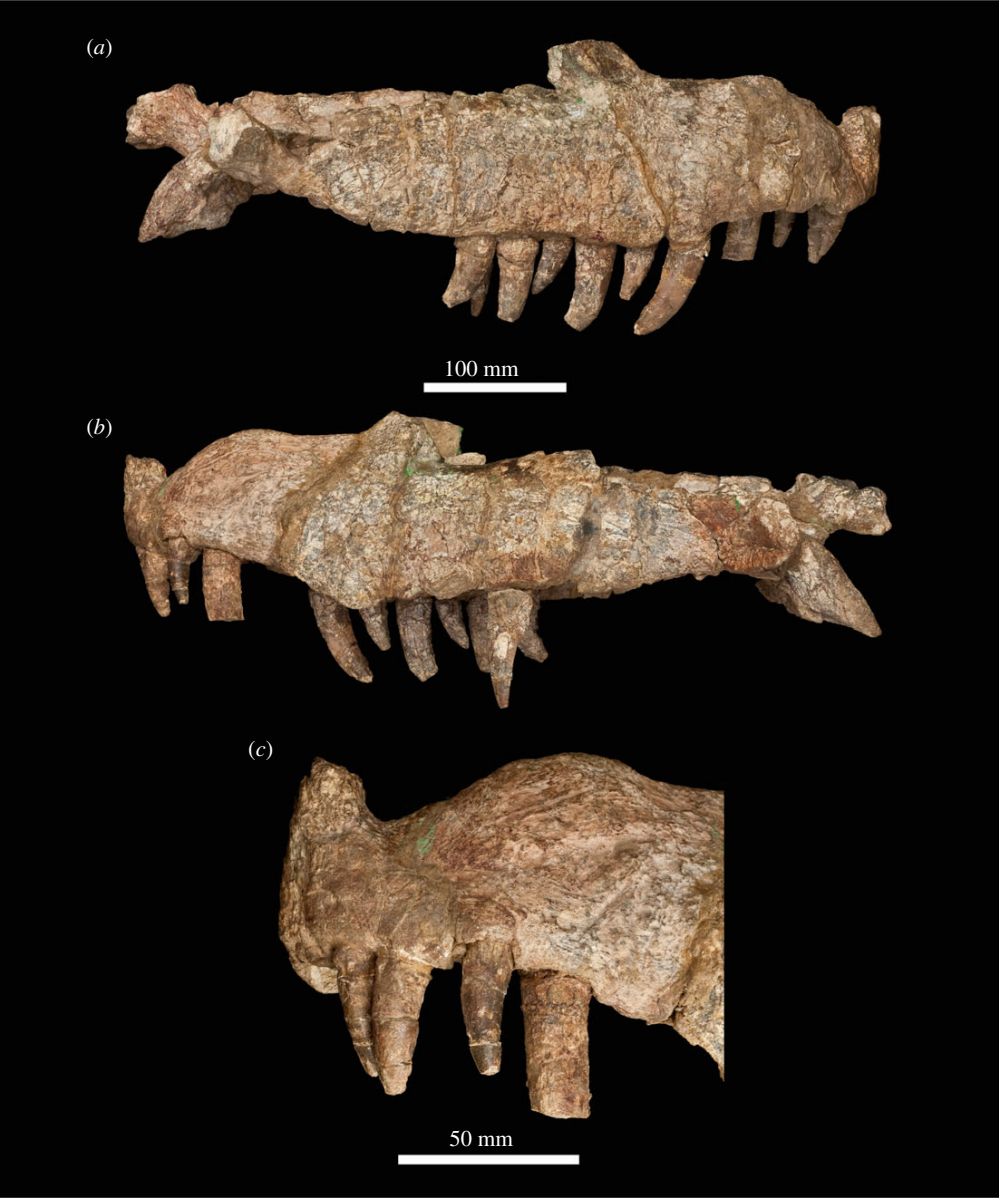
Photographs of the skull of NHMUK R36620, holotype of *Mambawakale ruhuhu*, in right lateral (*a*) and left lateral (*b*) views, with a close-up of the left premaxilla in lateral view (*c*).

**Figure 3.**
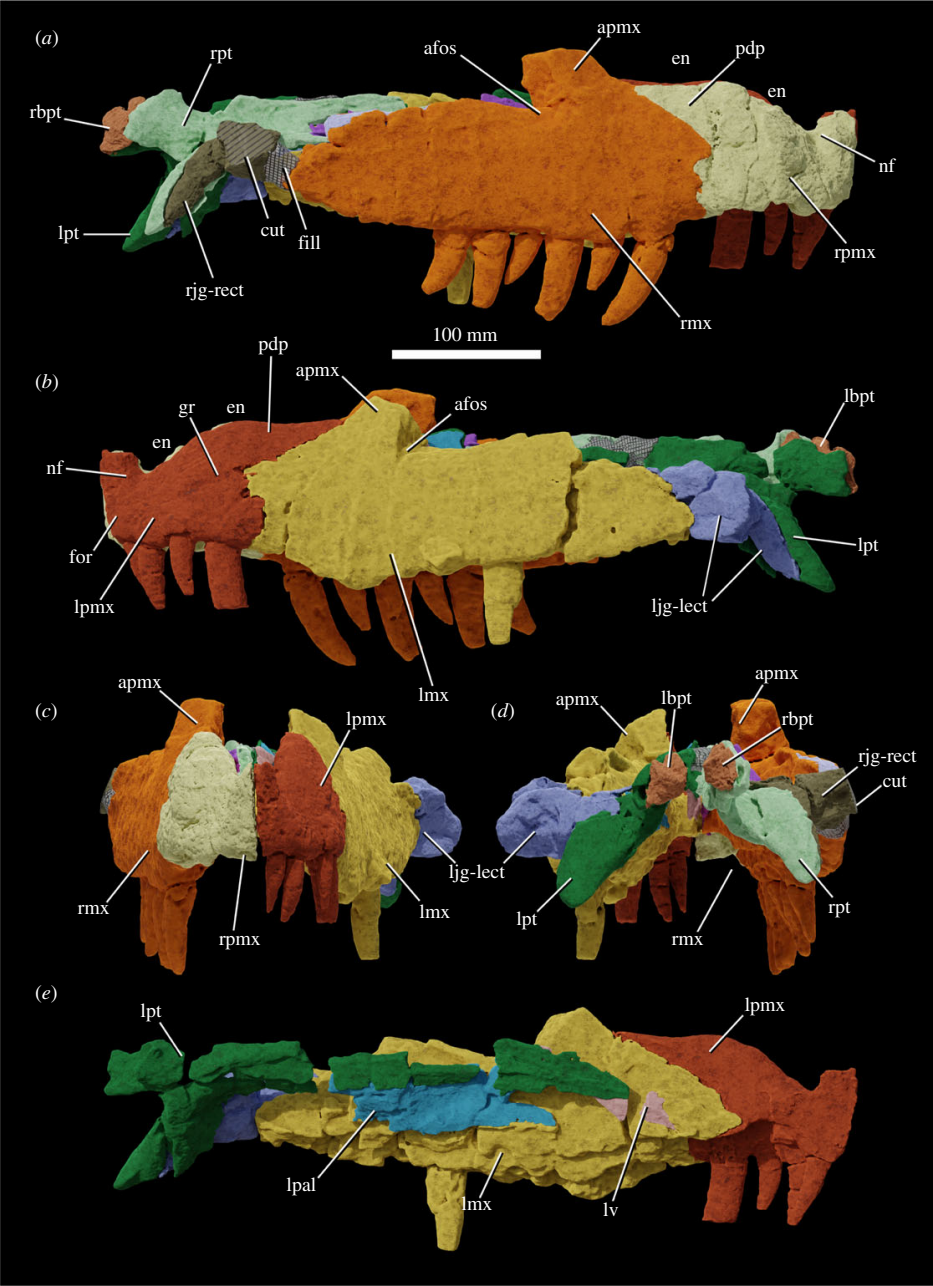
Segmentation based on CT data of the skull of NHMUK R36620, holotype of *Mambawakale ruhuhu*, in right lateral (*a*), left lateral (*b*), anterior (*c*) and posterior (*d*) views, with a medial view of the left side of the skull (*e*). Abbreviations: afos, antorbital fossa; apmx, ascending process of the maxilla; cut, small part of the fossil that was cut off and not included in the CT scan; en, external naris; fill, area of artificial repair; for, foramen; gr, groove on the lateral surface of the posterodorsal process of the premaxilla; lbpt, left basipterygoid process of the basisphenoid; ljg-lect, left jugal and left ectopterygoid; lmx, left maxilla; lpal, left palatine; lpmx, left premaxilla; lpt, left pterygoid; lv, left vomer; nf, narial fossa; pdp, posterodorsal process of the premaxilla; rbpt, right basipterygoid process of the basisphenoid; rjg-rect, right jugal and right ectopterygoid; rmx, right maxilla; rpmx, right premaxilla; rpt, right pterygoid.

**Figure 4.**
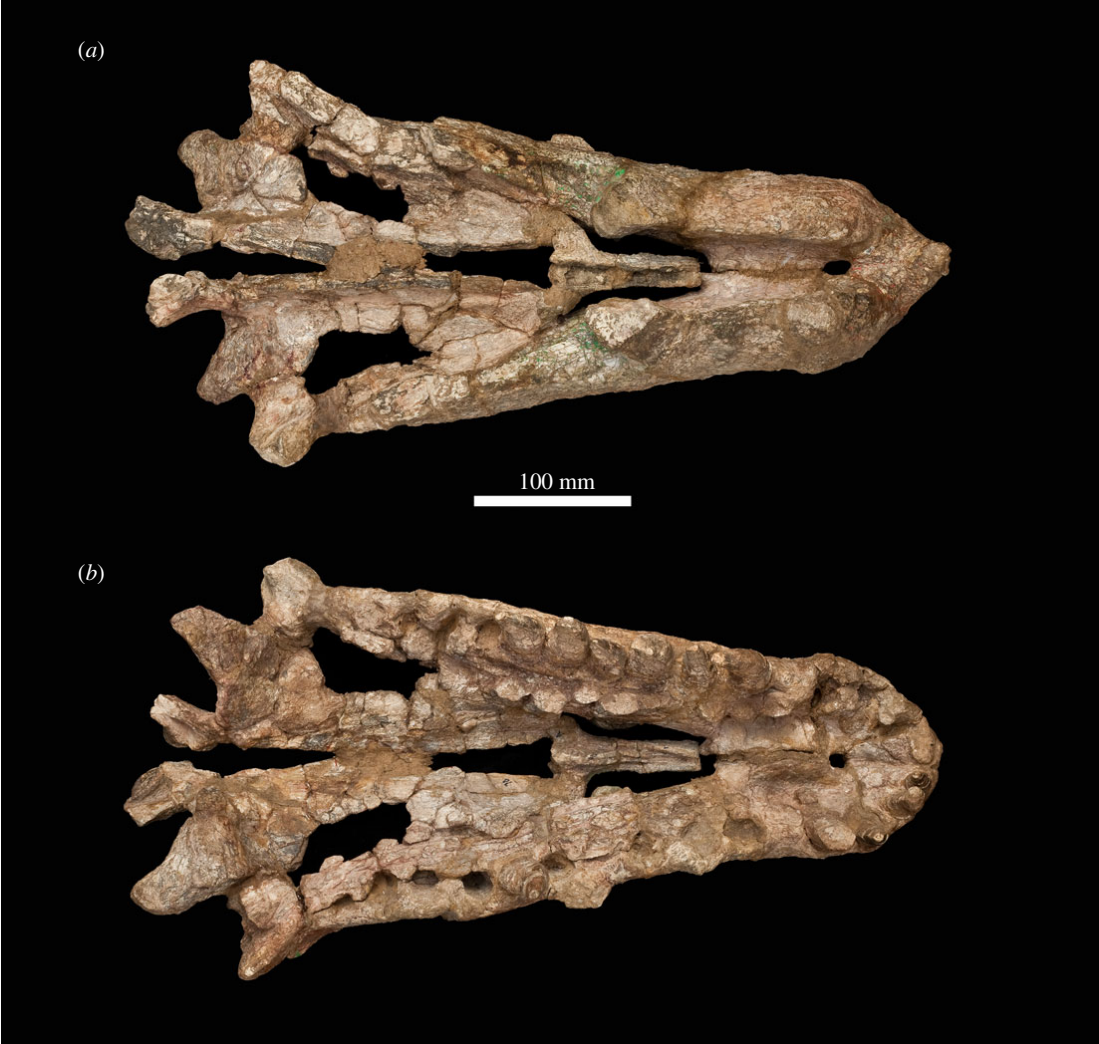
Photographs of the skull of NHMUK R36620, holotype of *Mambawakale ruhuhu*, in dorsal (*a*) and ventral (*b*) views.

**Figure 5.**
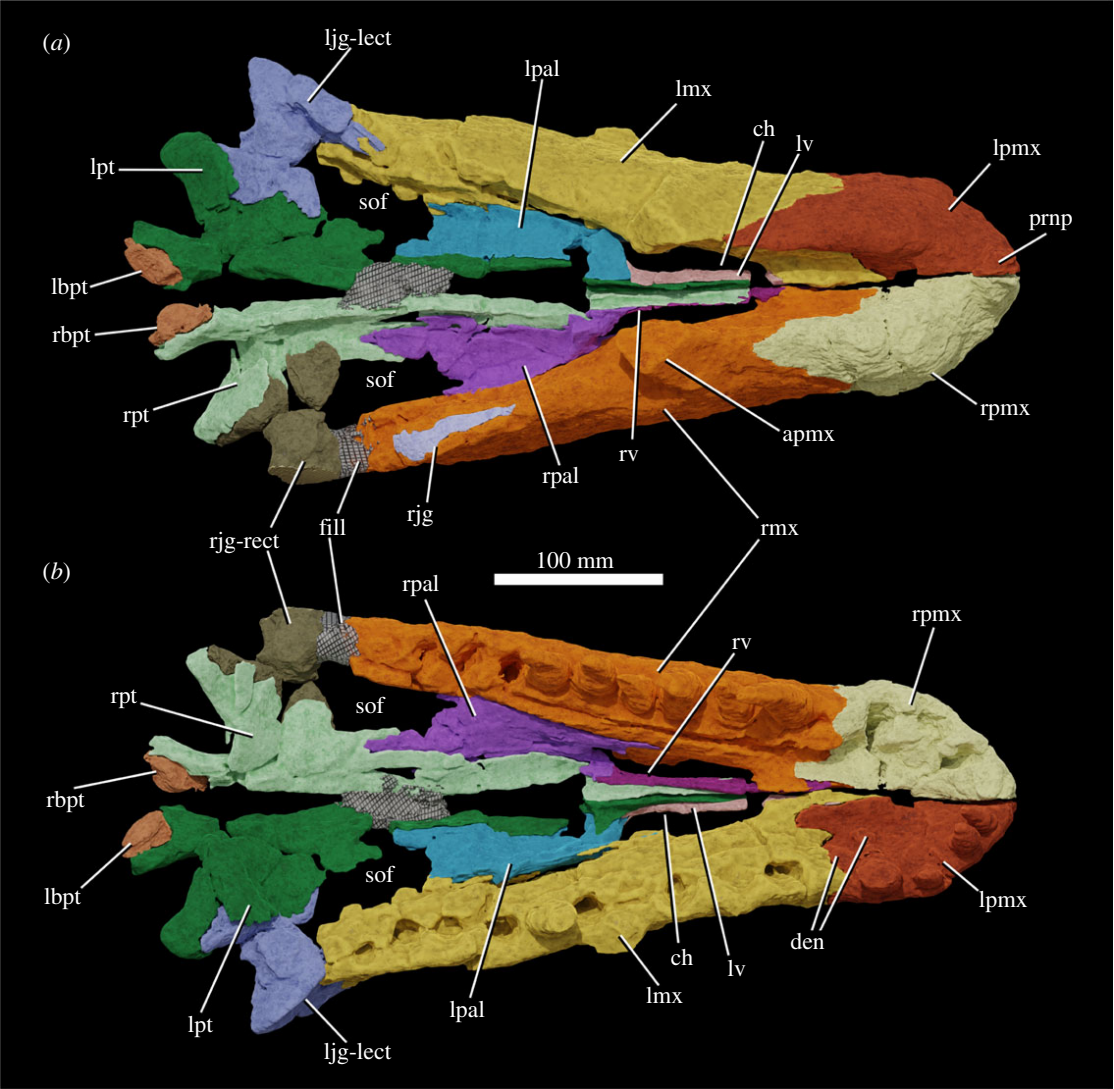
Segmentation based on CT data of the skull of NHMUK R36620, holotype of *Mambawakale ruhuhu*, in dorsal (*a*) and ventral (*b*) views. Abbreviations: apmx, ascending process of the maxilla; ch, choana; den, dentary tooth fragments attached to the premaxilla; fill, area of artificial repair; lbpt, left basipterygoid process of the basisphenoid; ljg-lect, left jugal and left ectopterygoid; lmx, left maxilla; lpal, left palatine; lpmx, left premaxilla; lpt, left pterygoid; lv, left vomer; prnp, prenarial process of the premaxilla; rbpt, right basipterygoid process of the basisphenoid; rjg, fragment of the right jugal; rjg-rect, right jugal and right ectopterygoid; rmx, right maxilla; rpal, right palatine; rpmx, right premaxilla; rpt, right pterygoid; rv, right vomer; sof, suborbital fenestra.

**Figure 6.**
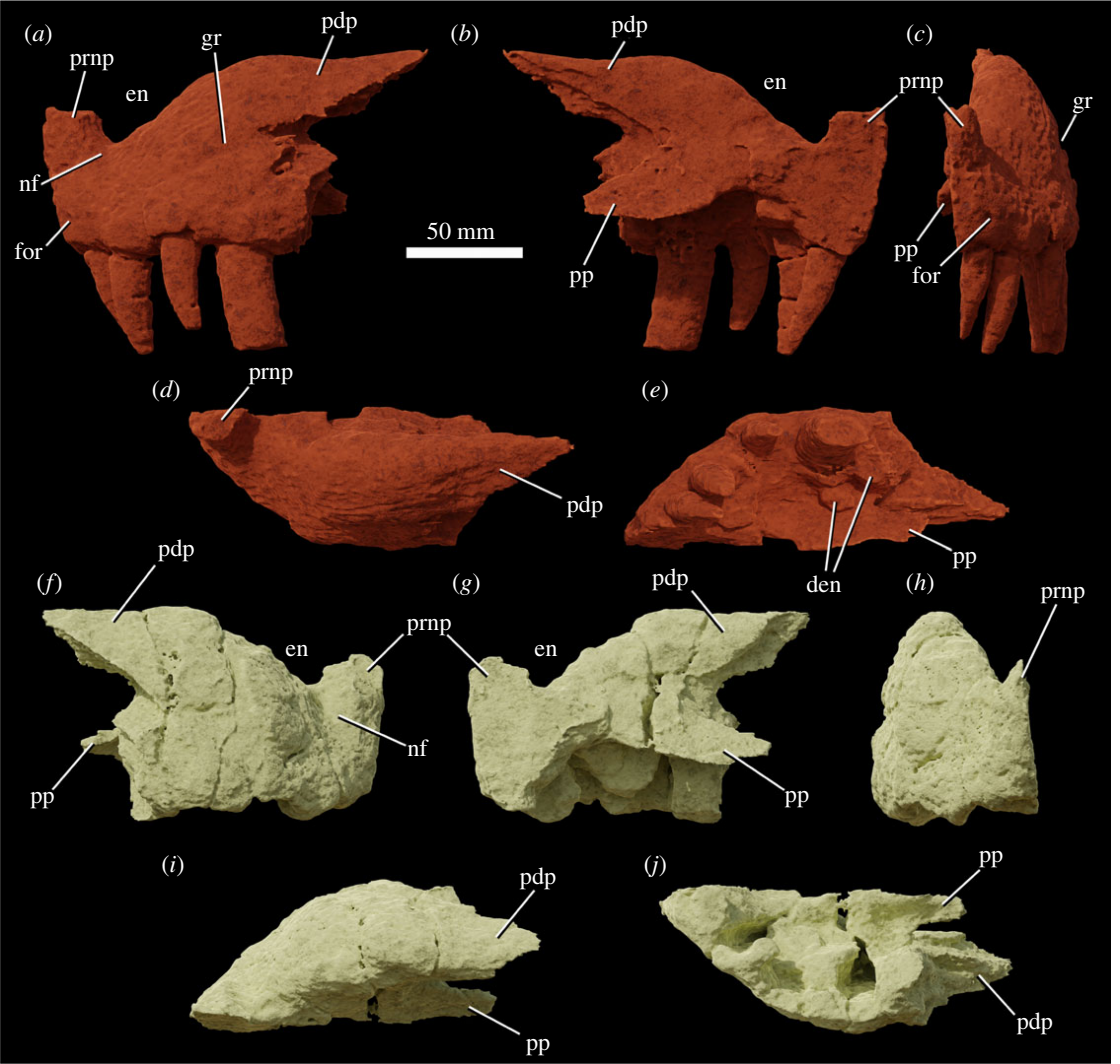
Premaxillae of NHMUK R36620, holotype of *Mambawakale ruhuhu*, reconstructed from CT data. Left premaxilla in lateral (*a*), medial (*b*), anterior (*c*), dorsal (*d*) and ventral (*e*) views. Right premaxilla in lateral (*f*), medial (*g*), anterior (*h*), dorsal (*i*) and ventral (*j*) views. Abbreviations: den, dentary tooth fragments; en, external naris; for, foramen; gr, groove on the lateral surface of the posterodorsal process; nf, narial fossa; pdp, posterodorsal process; pp, palatal process; prnp, prenarial process.

**Figure 7.**
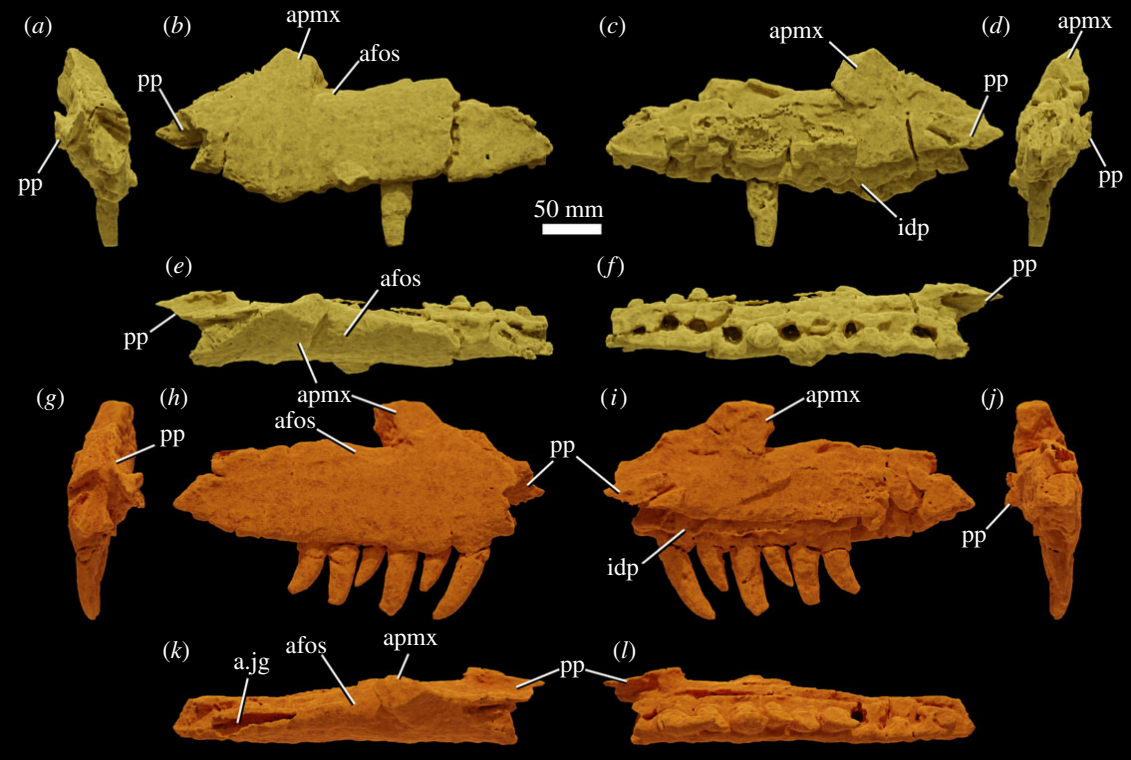
Maxillae of NHMUK R36620, holotype of *Mambawakale ruhuhu*, reconstructed from CT data. Left maxilla in anterior (*a*), lateral (*b*), medial (*c*), posterior (*d*), dorsal (*e*) and ventral (*f*) views. Right maxilla in anterior (*g*), lateral (*h*), medial (*i*), posterior (*j*), dorsal (*k*) and ventral (*l*) views. Abbreviations: afos, antorbital fossa; a.jg, articular surface for the jugal; apmx, ascending process of the maxilla; idp, interdental plates; pp, palatal process.

**Figure 8.**
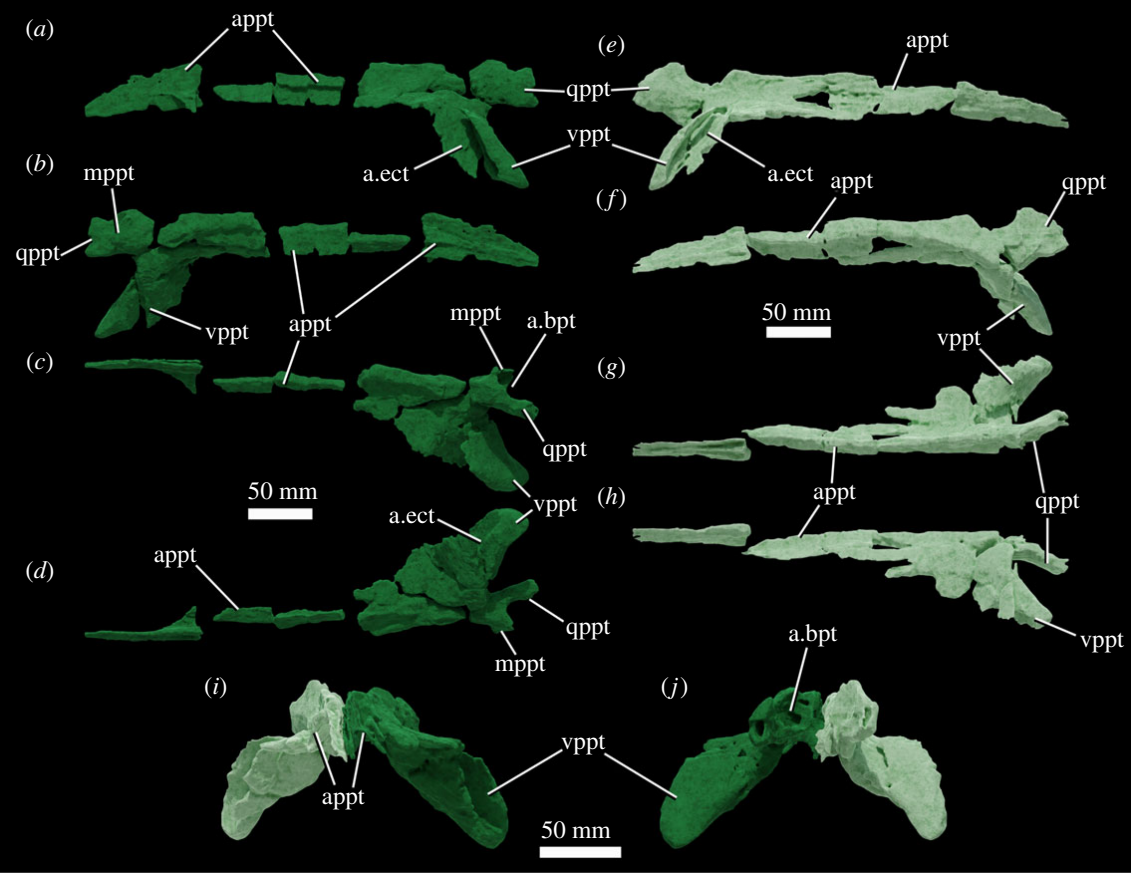
Pterygoids of NHMUK R36620, holotype of *Mambawakale ruhuhu*, reconstructed from CT data. Left pterygoid in lateral (*a*), medial (*b*), dorsal (*c*) and ventral (*d*) views. Right pterygoid in lateral (*e*), medial (*f*), dorsal (*g*) and ventral (*h*) views. Paired pterygoids in anterior (*i*) and posterior (*j*) views. Abbreviations: a.bpt, articular surface for the basipterygoid process of the basisphenoid; a.ect, articular surface for ectopterygoid; appt, anterior process of the pterygoid; mppt, medial process of the pterygoid; qppt, quadrate process of the pterygoid; vppt, ventral process of the pterygoid.

**Figure 9.**
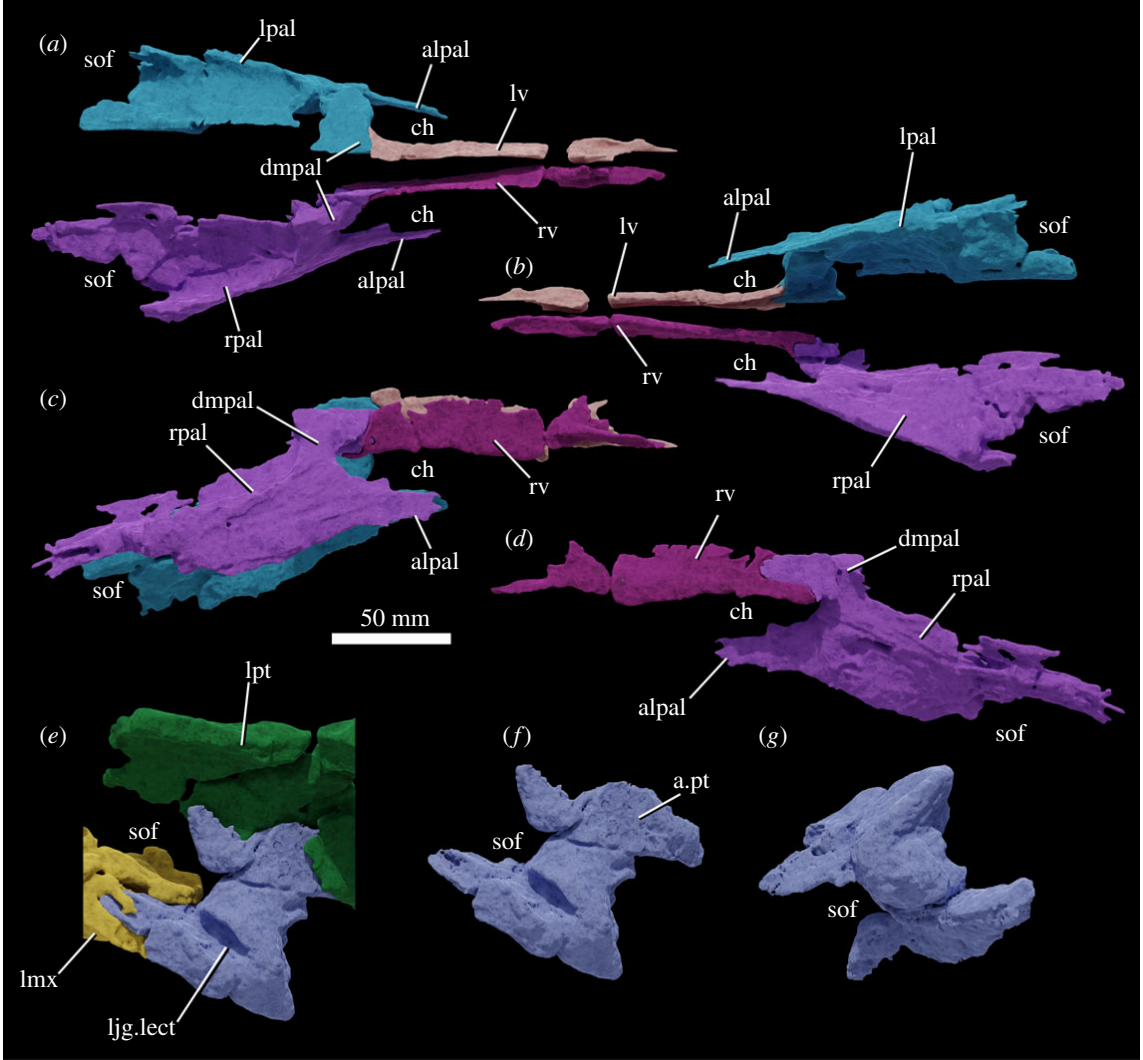
Vomers, palatines and left ectopterygoid-jugal of NHMUK R36620, holotype of *Mambawakale ruhuhu*, reconstructed from CT data. Articulated palatines and vomers in dorsal (*a*), ventral (*b*), and right lateral (*c*) views. Right palatine and vomer in medial (*d*) view. Dorsolateral view of the left ectopterygoid-jugal in articulation with the left pterygoid and maxilla (*e*). Left ectopterygoid-jugal in dorsolateral (*f*) and ventromedial (*g*) views. Abbreviations: alpal, anterolateral process of the anterior end of the palatine; a.pt, articular surface for the ventral process of the pterygoid; ch, choana; dmpal, dorsomedial process of the anterior end of the palatine; ljg-lect, left jugal and left ectopterygoid; lmx, left maxilla; lpal, left palatine; lpt, left pterygoid; lv, left vomer; rpal, right palatine; rv, right vomer; sof, suborbital fenestra.

**Figure 10.**
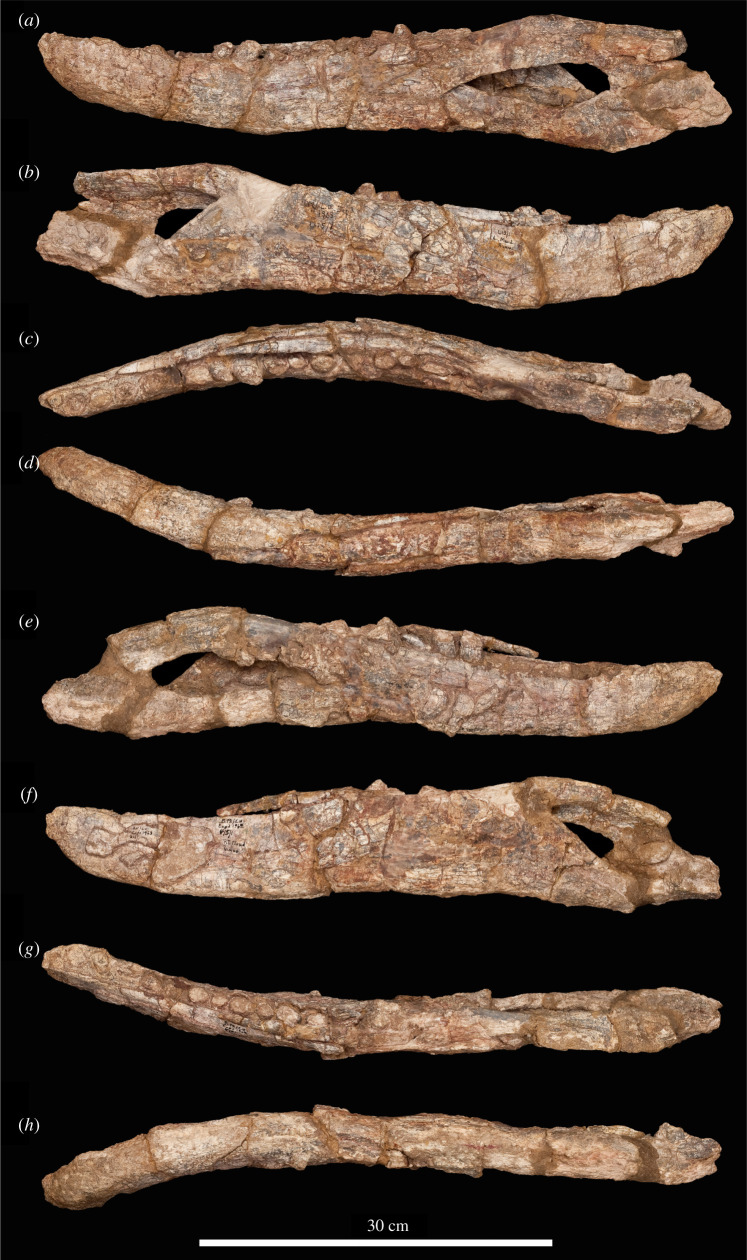
Photographs of the left and right hemimandibles of NHMUK R36620, holotype of *Mambawakale ruhuhu*. (*a–d*) The left hemimandible and (*e–h*) the right hemimandible in lateral (*a,e*), medial (*b,f*), dorsal (*c,g*) and ventral (*d,h*) views.

**Figure 11.**
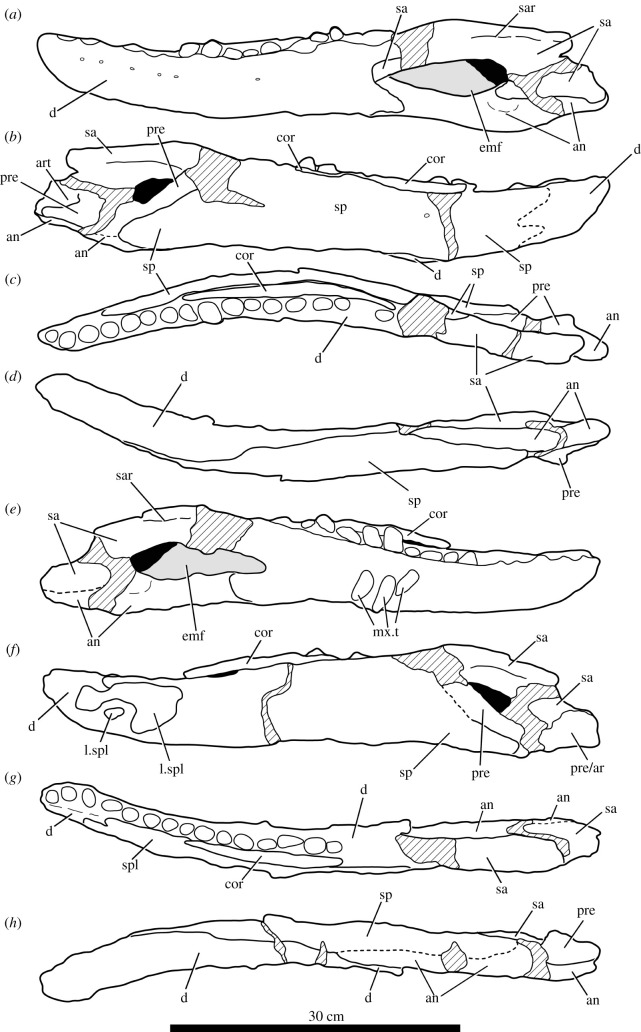
Line drawings of the left and right hemimandibles of NHMUK R36620, holotype of *Mambawakale ruhuhu*. (*a–d*) The left hemimandible and (*e–h*) the right hemimandible in lateral (*a,e*), medial (*b,f*), dorsal (*c,g*) and ventral (*d,h*) views. Cross-hatched areas indicate areas of artificial repair. Abbreviations: an, angular; art, articular; cor, coranoid; d, dentary; emf, external mandibular fenestra; l.spl, fragments of left splenial attached to right hemimandible; mx.t, maxillary teeth preserved attached to the dentary; pre, prearticular; pre/ar, prearticular/articular; sa, surangular; sar, surangular ridge; sp, splenial.

**Figure 12.**
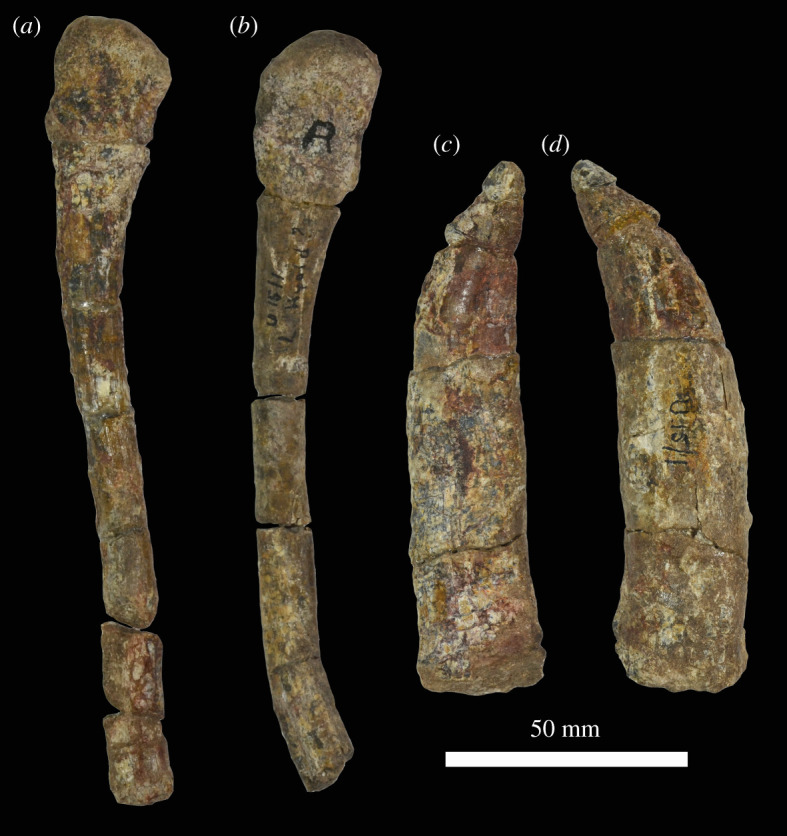
First ceratobranchials of the hyoid apparatus (*a,b*) and isolated tooth (*c,d*) of NHMUK R36620, holotype of *Mambawakale ruhuhu*.

The specimen was found with the hemimandibles in occlusion with the skull (figure 1), on a nearly flat erosional surface; the top of the skull including the braincase, quadrates, quadratojugals, much of the jugals, skull table and dorsal parts of the anterior skull elements were weathered away. The skull (figures 2–9) was separated from the mandible (figures 10 and 11), apparently during preparation, but in this process, many of the teeth of the left and especially the right hemimandibles were broken, such that their crown bases and roots are preserved within each hemimandible (figures 10 and 11), whereas the apices of the crowns are preserved adhered to the medial surfaces of the premaxillae and maxillae. Additional teeth and teeth fragments are also preserved separately (figure 12). Although the preserved skull elements are in articulation, the general preservation is poor. The external bone surface is firm but heavily cracked, with some larger breaks, and most sutures are impossible to identify with certainty on the exterior of the specimen, although these can be identified in many cases (with varying degrees of certainty) using CT data.

Only the ventral part of the margin of the external naris is preserved (figures 2, 3, 6 and 7, ‘en’), extending along most of the dorsal preserved edge of the premaxilla and the preserved anterior margin of the ascending process of the maxilla. This suggests that the naris was large and extended posteriorly to at least above the two most anterior maxillary teeth, but its exact dimensions and outline are unknown. A comparably large naris extending posteriorly above the anterior maxilla is also inferred to have been present in some other archosaurs (e.g. *Batrachotomus kupferzellensis*, [32]; *Arizonasaurus babbitti*, [50]; *Xilousuchus sapingensis*, [5]). In many archosaurs, the maxilla is excluded from the posterior border of the external naris, typically by contact between the nasal and the posterodorsal process of the premaxilla [6]. However, this contact is absent and the maxilla forms the posterior border of the external naris in some taxa (e.g. *Batrachotomus kupferzellensis*, [32]; *Arizonasaurus babbitti*, [50]; *Effigia okeeffeae*, [51]; *Xilousuchus sapingensis*, [5]).

#### 3.1.1. Premaxilla

Both premaxillae are preserved (figure 6), although their prenarial processes are broken away close to their bases. Contacts with the maxilla are unclear on the external surface but can be delimited with CT data (figure 3). Along the oral margin of the skull, premaxilla–maxilla contact occurs just posterior to the point where the snout is pinched inwards in ventral view (figure 5*b*), within a short edentulous region (diastema) separating the premaxillary and maxillary tooth rows. The CT data demonstrate that premaxilla–maxilla contact is extensive and immobile.

On the palatal surface, there is an oval opening anteriorly between the premaxillae, level with the boundary between the third and fourth tooth alveoli (figure 5*b*). This opens anteroventrally, explaining its more circular than oval appearance in ventral view. Each premaxilla bears a row of irregular pits on the palatal surface, positioned between the midline and the tooth row. These appear to receive the tips of the mandibular teeth.

The preserved part of the prenarial process of the premaxilla is slender and directed slightly anterodorsally (figure 6, ‘prnp’). The posterodorsal (= posterolateral; = maxillary) process of the premaxilla is elongate and tapers to a point distally (figures 3 and 6, ‘pdp’). This process has an interdigitating suture with the anterior and anterodorsal part of the maxilla and the tip of the process fits into a clear groove; together the maxilla and the posterodorsal process of the premaxilla form the smooth and rounded posterior and ventral borders of the external naris (figures 3, 6 and 7, ‘en’). An interdigitating suture between the premaxilla and the maxilla is rare and narrowly taxonomically distributed within Archosauria, occurring in only a few clades (phytosaurs, crocodylomorphs; [6]). The condition in *Mambawakale ruhuhu* is in contrast to that of paracrocodylomorphs (excluding Crocodyliformes) that almost all possess an unsutured and loose premaxilla–maxilla joint [32]. Furthermore, *M. ruhuhu* lacks an opening between the premaxilla and maxilla, unlike the condition in many paracrocodylomorphs where there is an opening ventral to the posterodorsal process of the premaxilla [32,46,52,53].

There is a well-developed narial fossa in the anteroventral corner of the naris (figure 6*a,f*, ‘nf’), though this merges with the main lateral surface of the premaxilla gradually rather than being delimited by a clear ridge. A foramen, probably neurovascular, is present on the anterolateral surface of each premaxilla, approximately 10 mm above the first tooth position (figure 6*a,c*, ‘for’). On the left premaxilla, there is a groove above the fourth tooth and extending posterodorsally onto the base of the posterodorsal process, strongly tapering and deepening as it does so (figure 6*a*, ‘gr’). This area of the right premaxilla is less well preserved. The posterodorsal process of the premaxilla is domed in lateral view above tooth positions 2–4, and is transversely very thick, particularly above tooth positions 3 and 4 (figure 6*d,i*). We interpret this as an autapomorphy of *Mambawakale ruhuhu*. The palatal process of each premaxilla articulates posteriorly with the palatal process of the maxilla (figures 5 and 6, ‘pp’). The maxillary palatal processes overlie the premaxillary processes dorsally and also separate the latter from one another on the midline (figure 5).

There are four tooth positions on each premaxilla (figures 5 and 6). The most posterior premaxillary tooth on both premaxillae is substantially larger than the other premaxillary teeth, and similar in size to those tooth positions in the anterior part of the maxilla. By contrast, premaxillary teeth 1–3 are sub-equal in size and substantially smaller than premaxillary tooth 4 or the anterior maxillary teeth. This strong size heterodonty of the premaxillary dentition represents an autapomorphy of *Mambawakale ruhuhu*. In other archosauriforms with toothed premaxillae, there is generally limited heterodonty in the premaxillary teeth [6,37]. The premaxillary teeth are transversely compressed. Premaxillary teeth 2–4 are recurved, whereas curvature is less in the first tooth. The third premaxillary tooth on the left side is the best preserved and has fine serrations along mesial and distal surfaces. Denticle counts are available for the mesial margin of the first premaxillary tooth (three per mm) and the mesial (2.5 per mm) and distal (two per mm) margins of the third premaxillary tooth [21]. A short diastema is present between the premaxillary and maxillary tooth rows.

#### 3.1.2. Maxilla

Both maxillae are preserved, but the ascending process of each has broken away close to its base (figures 2–5 and 7, ‘apmx’). The posterior end of each bone is also somewhat broken along its posterodorsal margin. The ventral margin of the maxilla is convex in lateral view, with the maximum convexity lying below the anterior part of the ascending process. The premaxillary tooth row is set dorsal to the majority of the maxillary tooth row (figures 2 and 3). The preserved bases of the ascending processes of the maxillae are oriented posterodorsally and are positioned above the second and third maxillary alveoli. The cross section of each ascending process is triangular in dorsal view (figure 7*e,l*, ‘apmx’). A lateral ridge on the ascending process forms the anterior border of the antorbital fossa (figure 7*b,i*, ‘afos’), but this is weakly developed as in *Prestosuchus chiniquensis* [54] and *Saurosuchus galilei* [55]. The antorbital fossa is relatively deep on the posterolateral surface of the ascending process and continues onto and posteriorly along the horizontal process, fading out above the sixth maxillary tooth, similar to *P. chiniquensis* [54] and *S. galilei* [55]. The external surfaces of the maxillae are poorly preserved but are generally smooth without any notable rugosity. On the left side, foramina are visible on the external surface of the maxilla, towards the dental margin above the second tooth and between the second and third teeth. On the right side, a row of foramina are visible, and these have grooves that extend ventrally from them towards the dental margin. The posterior process of the maxilla does taper posteriorly, but remains dorsoventrally tall for much of its length, like that of *P. chiniquensis* [54] and *S. galilei* [55].

Above the sixth tooth position, a posteriorly opening foramen on the medial surface of the maxilla, slightly ventral to the dorsal margin of the horizontal process, is perhaps for the maxillary nerve and associated blood vessels. A groove extends posteriorly from this foramen, immediately ventral to and overhung by the ventral margin of the antorbital fenestra.

The maxillae are straight in dorsal or ventral view and diverge from one another towards their posterior ends (figures 4 and 5). The palatal processes arise medial to the first maxillary tooth and extend anteromedially (figures 5*b* and 7, ‘pp’). They contact one another on the midline, overlie the palatal processes of the premaxillae anteriorly, and are contacted posteriorly by the anterior ends of the vomers. There is a low step on the medial surface of each maxilla, sub-parallel to and approximately 10 mm above the tooth-bearing margin (figure 7*c,j*). Interdental plates are immediately ventral to this step and have a sub-pentagonal outline (figure 7*c,j*, ‘idp’). The contact of the maxilla with the palatine occurs approximately 10 mm dorsal to the low step. It is unclear whether there was contact between the ectopterygoid and maxilla immediately posterior to the tooth row due to poor preservation in this area on both sides of the skull.

The tooth count is 10 for both maxillae. All the maxillary teeth are large with little variation in size of preserved teeth and/or alveoli, although gentle reduction in size occurs over the last 3–4 positions. Both mesial and distal denticles are present. Denticle counts are available for five of the teeth, with two per mm on the mesial margin and two to three per mm on the distal margin [21]. Implantation is thecodont.

#### 3.1.3. Jugal

Small, mostly anteroventral parts of the jugals appear to be preserved (figures 3, 5 and 9), although the boundary between the ectopterygoid and jugal cannot be clearly delimited on either side, even with CT data. Parts of the jugal clearly extend anteriorly into the slot on the posterodorsal edge of the maxilla (figure 7*k*, ‘a.jg’), terminating above maxillary tooth position 7. If the ectopterygoid contact with the medial surface of the jugal occurred ventral to the postorbital process of that bone, the posterior extent of the antorbital fenestra can be partially constrained. It is unlikely that the antorbital fenestra extended posterior to the eighth maxillary tooth alveolus, and it may have been even shorter anteroposteriorly. This means that the anteroposterior length of at least the lower part of the antorbital fenestra may have been less than that of the external naris, similar to the condition in some other Triassic archosaurs with enlarged nares, as noted above.

#### 3.1.4. Palate - general

The palate is fairly complete, with substantial parts of the vomers, palatines, pterygoids and ectopterygoids all preserved (figures 4 and 5). The choanae are narrow elliptical openings medial to the first four maxillary teeth (figures 4 and 5, ‘ch’). The oval- to kidney-shaped suborbital fenestrae are similar in length to the choanae (figures 4 and 5, ‘sof’). No palatal teeth are present on any of the bones.

#### 3.1.5. Pterygoid

The pterygoids are tetraradiate (figures 4, 5 and 8). As preserved, they approach one another but are not in contact along the midline. Their plate-like anterior processes (figure 8, ‘appt’) are arched upwards towards the midline and form most of the medial margins of the suborbital fenestrae. Anterolaterally, they form an extensive contact with the palatines, and are dorsally overlapped by the latter bones. The tips of the anterior processes reach far anteriorly, separating the vomers at the midline and terminating just short of the palatal processes of the maxillae. The transversely compressed quadrate processes of the pterygoids (figure 8, ‘qppt’) are both broken at their bases, shortly posterior to their contacts with the basipterygoid processes of the basisphenoid. A short medial process (figure 8, ‘mppt’), only preserved on the left side, extends medially from the base of the quadrate process, and together the bases of the medial and quadrate processes form an articular surface for the basipterygoid process of the basisphenoid (figure 8, ‘a.bpt’). The ventral processes of the pterygoids extend laterally and posteroventrally and have a sub-triangular outline with a rounded distal end in dorsal view (figure 8, ‘vppt’). The ventral process forms the posteromedial margin of the suborbital fenestra. A deep concavity on the anteroventral surface of the ventral process is the articulation surface for the ectopterygoid (figure 8, ‘a.ect’).

#### 3.1.6. Palatine

Most parts of the palatines are preserved (figures 4, 5 and 9). They form the posterior and posterolateral margins of the choanae, the anterior margins of the suborbital fenestrae, and a long lateral contact with the maxilla, extending medial to maxillary tooth positions 3–9. Medially, they form extensive anteroposteriorly oriented contacts with the pterygoids, dorsally overlapping the latter bones, and anteromedially they contact the vomers. The main body of the palatine is a dorsoventrally compressed plate with a transversely concave dorsal surface, and a broadened posterior end. The lateral margin of the main body is expanded dorsoventrally where it articulated with the maxilla. At its anterior end, the palatine divides into two processes: a transversely compressed anterolateral process that articulates with the maxilla (figure 9*a–d*, ‘alpal’) and forms the posterolateral margin of the choana, and a dorsomedial process that articulates medially with the posterior end of the vomer and forms the posterior margin of the choana (figure 9*a–d*, ‘dmpal’).

#### 3.1.7. Vomer

The vomers are sub-vertical sheets that are transversely compressed and separated from one another on the midline by the anterior tips of the anterior processes of the pterygoids (figures 4, 5 and 9). They form the medial margins of the choanae. Anteriorly their tips contact the palatal processes of the maxillae and posteriorly they are contacted laterally by dorsomedial processes of the palatines.

#### 3.1.8. Ectopterygoid

The ectopterygoids are arched bar-like bones that articulate laterally with the medial surfaces of the jugals, immediately posterior to the posterior ends of the tooth rows (figures 4, 5 and 9). The contacts with the jugals cannot be clearly identified, even in CT data. The ectopterygoid forms the lateral portion of the posterior margin of the suborbital fenestra, and medially the bone is anteroposteriorly expanded and articulates with and underlies the anterior surface of the ventral process of the pterygoid.

#### 3.1.9. Basisphenoid

Small parts of the distal ends of the basipterygoid processes of the basisphenoid are preserved in articulation with the pterygoids (figures 2–5, ‘lbpt’, ‘rbpt’) but provide little anatomical data.

### 3.2. Mandible

Both hemimandibles are mostly complete (figures 10 and 11), although the articular and retroarticular region of each is badly damaged and/or incomplete. Preservation is generally poor, and sutures are difficult or impossible to identify. Together, the hemimandibles form an anteroposteriorly elongate symphysis that extends as far as tooth position 8, giving the anterior end of the entire mandible a transversely narrow appearance in dorsal view. A similarly elongate symphysis was considered a synapomorphy of Ornithosuchidae by Sereno [56], but also occurs in phytosaurs, crocodyliforms, *Shuvosaurus inexpectatus* and *Effigia okeeffeae* [6]. At the end of the symphysis, there is a distinct inflexion point where the hemimandibles diverge from one another posteriorly (figures 10*c*,*g* and 11*c,g*). There is a large, dorsoventrally shallow, external mandibular fenestra (figures 10*a,e* and 11*a,e*, ‘emf’), the border of which is rounded posteriorly and tapered anteriorly.

#### 3.2.1. Dentary

The dentary is largely complete on each side. In dorsal view, it is laterally concave due to the inflexion point noted above (figures 10*c,g* and 11*c,g*). In lateral view, it is dorsoventrally shallow and tapers anteriorly with no dorsoventral expansion at the anterior end (figures 10*a,e* and 11*a,e*). On the lateral surface, there is an anteroposteriorly extending groove just above midheight. Foramina are evident within this groove, although the exact number is obscured by poor preservation of surface detail. Additional foramina are present on the ventral portions of the lateral surface of the dentary at its anterior end but are also poorly preserved. At its posterior end the dentary bifurcates into posterodorsal and posteroventral processes, which frame the anterior end of the external mandibular fenestra in lateral view (figures 10*a,e* and 11*a,e*). The posteroventral process laterally overlaps the angular. The contact of the posterodorsal process with the surangular is not preserved—this area is broken and reconstructed on both sides.

There appear to be at least 15, and possibly 16, tooth positions on left and right dentaries, although the exact count is unclear because of damage to the posterior ends of the tooth rows. Only the roots or broken bases of the crowns are preserved *in situ* in the dentary in nearly all cases, although a distorted but complete crown is preserved in the 13th tooth position on the right side. The best-preserved dentary crown apices are from the right dentary, preserved attached to the medial surface of the right maxilla. The best preserved of these are ziphodont, being transversely compressed and recurved, with fine denticles clearly present along mesial and distal carinae.

#### 3.2.2. Coronoid

There is an elongate, strap-like to sub-cylindrical coronoid bone (figures 10 and 11, ‘cor’), slightly bowed along its length, which extends from medial to the distal margin of tooth position 8 (immediately posterior to the termination of the mandibular symphysis) through to the posterior end of the tooth row. This bone is dorsoventrally narrow with a dorsoventrally convex medial surface, and as preserved is hidden in medial view on the left side by the splenial posterior to tooth position 12. By contrast, the posterior part of the coronoid is visible in medial view on the right hemimandible. The coronoid is a generally poorly studied element in Triassic archosauriformes, often not or poorly preserved or disarticulated from other mandibular elements. An elongate coronoid, like that of *Mambawakale ruhuhu*, is probably plesiomorphic for Archosauriformes (though coronoid length and/or shape has generally not been included as a character in recent phylogenetic analyses, e.g. [6,37]), being found in, for example, erythrosuchids [2,57], *Euparkeria capensis* [58], proterochampsids [59], at least some avemetatarsalians [60], the loricatans *Etjosuchus recurvidens* [61] and possibly *Saurosuchus galilei* [55]. Shorter coronoids are present in some archosaurs, such as *Prestosuchus chiniquensis* [62]. An element similar to the elongate coronoid of *Mambawakale ruhuhu* is also known in several dinosaurs, where it has sometimes been referred to as a ‘supradentary’, but the homology of this element across archosauromorphs remains unclear [63].

#### 3.2.3. Splenial

The splenial extends from medial to the fourth dentary alveolus posteriorly almost to the base of the posterior margin of the adductor fossa, covering most of the medial surface of the dentary, including the Meckelian groove (figures 10*b,f* and 11*b,f*, ‘sp’). The medial surface of this bone is heavily fractured and poorly preserved. On the left side, there appears to be a medium-sized foramen (*ca* 4 mm in diameter) on the dorsal half of the bone (figure 11*b*), below the 10th tooth position, but preservation is too poor to determine if this is also present on the right splenial.

#### 3.2.4. Surangular

The surangular has a convex, slightly angled dorsal margin in lateral view, and formed the majority of the dorsal and at least half of the posterior margin of the external mandibular fenestra (figures 10 and 11, ‘sa’). The precise position and nature of its anterior contact with the dentary are unknown due to damage. Both left and right surangulars are also damaged posteriorly, obscuring details of their contributions to the articular and postarticular regions and their contacts with the angulars, although they overlap the latter bones laterally, and contributions to the articular and postarticular regions. There is a low ridge on the lateral surface of the surangular (figures 10*a,e* and 11*a,e*, ‘sar’), and above this the dorsal part of the bone is inflected medially, to form a mostly dorsally and slightly laterally facing, slightly concave platform that would have met the upper jaw when the mouth was closed (figures 10*c,g* and 11*c,g*).

#### 3.2.5. Angular

The angular forms the ventral margin of the posterior third of the mandible, and its ventral margin is convex in lateral view (figures 10 and 11, ‘an’). The posterior end of the left angular is damaged but appears to be nearly complete on the right angular, suggesting that the retroarticular process, although damaged, may have been relatively short. Anteriorly, the angular wedges between the posteroventral process of the dentary and the splenial. The dorsal margin of the angular is concave and forms the entire ventral border of the external mandibular fenestra. There is a shallow concavity on the lateral surface of the bone, adjacent to the posteroventral corner of the external mandibular fenestra.

#### 3.2.6. Prearticular

The prearticular is preserved on the medial surface of the hemimandible and is visible through the external mandibular fenestra in lateral view (figures 10 and 11, ‘pre’). Its anterodorsal extension is damaged on both hemimandibles. It is medially overlapped by the posterior end of the splenial, and ventrally its lateral surface contacts the angular. The prearticular appears to form the medial surface of at least the base of the seemingly short retroarticular process, and the anterior and medial margins of the cup for the articular.

#### 3.2.7. Articular

The articular region is very poorly preserved, and although small parts of the articular are probably present, little can be determined about their morphology (figures 10 and 11, ‘art’). As far as can be determined, no part of the mandibular cotyle is preserved.

### 3.3. Hyoid apparatus

Two elements of the hyoid apparatus are preserved (figure 12*a,b*), and a note associated with the specimen indicates that at least one of these was found appressed to the posterior part of the right hemimandible. The two bones are approximately symmetrical and probably represent paired elements. They are slender bones that are sigmoidally curved along their lengths as preserved, and both are incomplete at the narrower ends. In both elements, the complete end of the bone is expanded with a triangular to oval cross section. They probably represent the first ceratobranchials, which are the dominant elements of the hyoid apparatus in modern reptiles [64], and which are also known in several extinct archosaurs [64–66].

### 3.4. Cervical vertebrae

**Figure 13.**
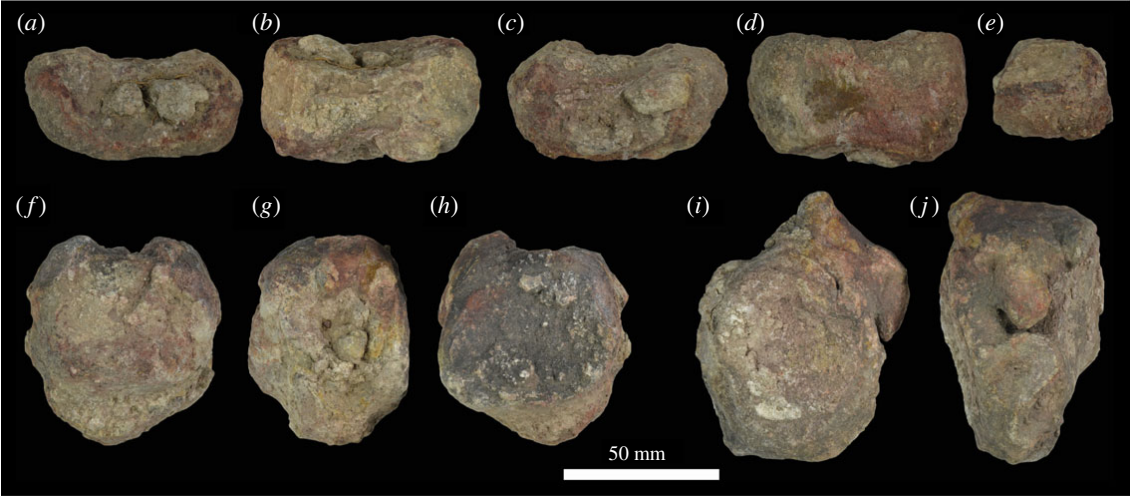
Anterior cervical vertebrae of NHMUK R36620, holotype of *Mambawakale ruhuhu*. Atlantal intercentrum in anterior (*a*), dorsal (*b*), posterior (*c*), ventral (*d*) and left lateral (*e*) views. Axis in anterior (*f*), right lateral (*g*) and posterior (*h*) views. Cervical 3 in anterior (*i*) and left lateral (*j*) views.

Parts of the first three cervical vertebrae are preserved (figure 13) but have not been prepared. The intercentrum of the atlas is preserved (figure 13*a–e*), but the neural arches are absent. The intercentrum has concave anterior and posterior faces, the former being more deeply concave. The element has a flat broad ventral surface and a concave dorsal margin in anterior view. The dorsolateral corners of the element are bevelled to form articular surfaces for the neural arches. The neural arch of the axis (figure 13*f–h*) is almost entirely broken away, although small parts of the base are present on the left side. The anteroposterior length of the axis centrum is slightly shorter than its dorsoventral height. Its anterior surface has a flat upper part and a saddle-shaped ventral part where it articulated with the missing axial intercentrum. The posterior articular surface of the centrum is deeply concave. The lateral surface of the centrum is also deeply concave anteroposteriorly, although much of this concavity is infilled with sediment. There are no distinct preserved articular surfaces for the axial ribs. The posteroventral margin of the centrum is strongly bevelled ventral to the posterior articular face, and this in combination with a similar bevelled surface on the anteroventral margin of the third cervical suggests the presence of an intercentrum between the axis and cervical 3. This is potentially a unique condition among crown archosaurs [6], although the presence of intercentra in *M. ruhuhu* cannot be confirmed without more complete material, and the condition is not yet known with certainty in all crown archosaurs. The ventral margin of the centrum has a broad longitudinal ridge, rather than a narrow keel.

Cervical 3 is missing most of the posterior and posteroventral part of the centrum, almost all the right neural arch, and most of the left neural arch (figure 13*i,j*). Overall, centrum proportions appear to be similar to those of the atlas—i.e. slightly taller dorsoventrally than long anteroposteriorly. The concave anterior articular face of the centrum is slightly taller dorsoventrally than wide. Ventral to the articular face, the anteroventral corner of the centrum is strongly bevelled, sloping from anterodorsal to posteroventral. The articular surfaces of the left parapophysis and diapophysis are damaged, and these apophyses are not exposed on the right but are positioned very close to one another on the anterior half of the centrum. The diapophysis overhangs a deep concavity on the lateral face of the centrum. At least anteriorly, the ventral margin of the centrum appears to have a midline ridge bounded by a pair of concavities.

### 3.5. Manus

**Figure 14.**
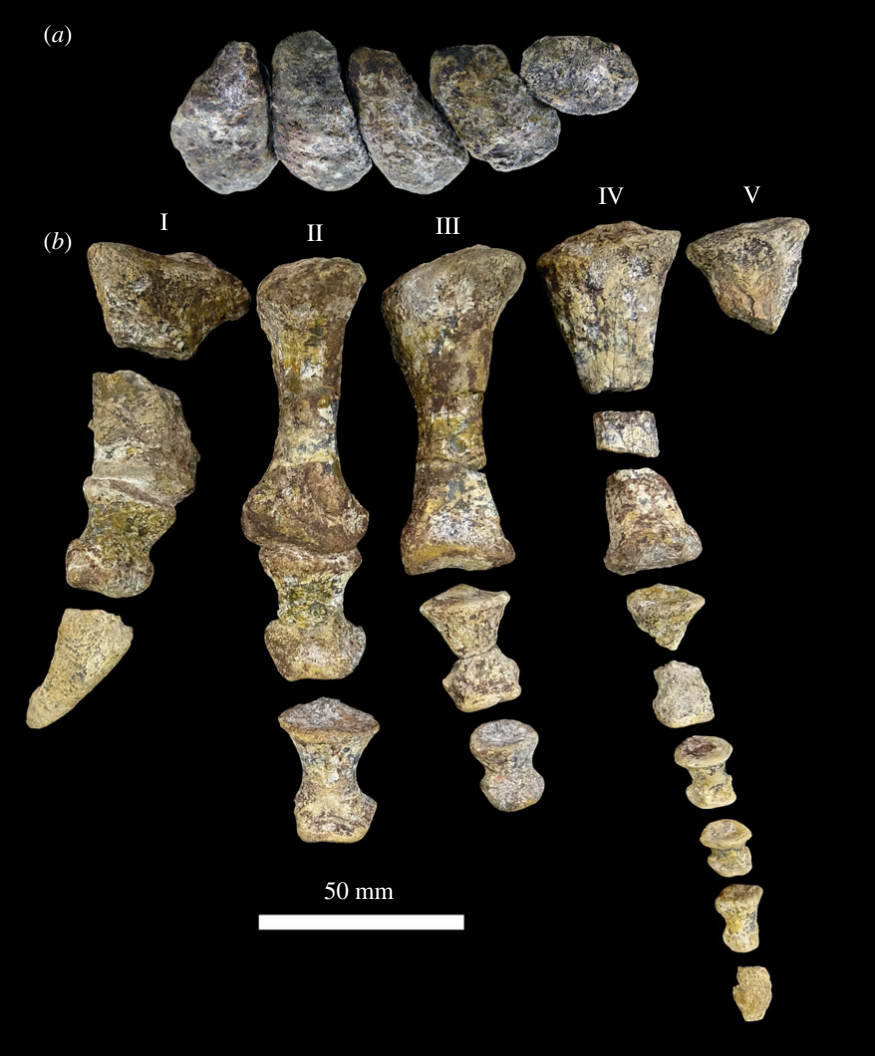
Left manus of NHMUK R36620, holotype of *Mambawakale ruhuhu*. Metacarpals in articulation in proximal view (*a*), dorsal surface directed to the bottom of the image. Manus in dorsal view (*b*). Digits ordered from I (left) to V (right) in both *a* and *b*.

A fairly complete left manus (figure 14) was preserved with the holotype of *Mambawakale ruhuhu* and we refer the manus to the same individual based on proximity and its consistent size with the skull. The manus includes metacarpals I to IV, the proximal portion of metacarpal V, phalanges of the first digit including an ungual, the first two phalanges of digits II and III, and phalanges 1–4 of digit IV as well as a potential ungual. Unfortunately, there are no field or preparation notes that describe the articulation or association of the manus in the field. However, some of the metacarpals remain articulated with their first phalanx and, furthermore, the phalanges were placed into boxes with a matching metacarpal after preparation; it appears that much of the manus was articulated and that care was taken to keep the associations among the digits.

Metacarpal I is broken into proximal and distal portions; the distal portion remains in articulation with phalanx I1. Metacarpal II also remains in articulation with its first phalanx, while metacarpal IV is broken in three pieces (proximal portion, mid-shaft, and distal end). The first phalanx of digit IV is also broken midway through the shaft.

Metacarpals III and IV are of similar length and the longest, followed by metacarpals II and I. Although incomplete, metacarpal V would probably have been the shortest based on the size of the proximal portion and the tapering of the midshaft (table 1). All the metacarpals are proximodistally elongate relative to their widths, with transversely expanded proximal and distal ends of which the proximal expansion is the greatest, and transversely constricted shafts that are at their minimum diameter distal to the mid-shaft. When in articulation, the proximal ends of the metacarpals are imbricated and form a tightly compressed arch (figure 14*a*). A tongue-and-groove articulation between metacarpal I and metacarpal II is not present, unlike the condition in *Postosuchus alisonae* [67,68].

**Table 1.** Measurements (in millimetre) of the manus of *Mambawakale ruhuhu* NHMUK R36620. Abbreviations: pdL, proximal-distal length; pW, maximum proximal width (medial lateral); pD, maximum proximal depth (dorsal ventral); sW, minimum shaft width; sD, minimum shaft depth; dW, maximum distal width; dD, distal depth.

	pdL	pW	dW	sW	sD	pD	dD
metacarpal I^a^		38	25			23.5	17
phalanx I1	21	21	19.5	14	10	18	13
phalanx I2 (ungual)	35	15				18	
metacarpal II	65	29	23	12.5	10.5	37	20
phalanx II1	34	21	22	13	10	19	12
phalanx II2	31.5	22	18	10.5	8	18	12
metacarpal III	72	24.5	24	10.5	11	36	20
phalanx III1	27	19.5	16.5	8.5	7	9	11
phalanx III2	19	15	13	9	5.5	12	8
metacarpal IV^a^		22	21			30	20
phalanx IV1^a^	27^b^	15	11			11	7
phalanx IV2	15	11.5	9	6	4	9.5	4
phalanx IV3	10	10	8	6	4	7	5
phalanx IV4	13	9	6	5	2	6	3
phalanx IV5 (ungual)^a^	14						
metacarpal V^a^		17				26	

^a^Element broken/damaged.

^b^Measurement estimated.

In dorsal view, metacarpal I is the most robust element, and its shaft is dorsoventrally flattened with a sub-elliptical cross section. The proximal end of the metacarpal is sub-triangular in proximal view. The distal articulation surface is sub-rectangular in outline and asymmetrical, with the larger lateral condyle separated from the medial condyle by a shallow fossa. The ligament pit of the lateral condyle is deeper than that of the medial one. A pronounced extensor ligament pit is located on the dorsal surface of the distal end of the metacarpal.

Metacarpals II and III are very similar in shape, being more elongate and slenderer than metacarpal I, with more circular shafts in cross section. In proximal view, the outline of metacarpals II and III is sub-elliptical with gently concave ventrolateral margins. The distal ends are also asymmetrical, with larger medial condyles with relatively flat medial surfaces, while lateral condyles have more pronounced collateral ligament pits. The lateral ligament pit is much deeper in metacarpal II than in metacarpal III. As in metacarpal I, there is a fossa distally separating the two condyles, the outline of the distal surface is sub-rectangular and ventrally concave, and there is a pronounced extensor ligament pit on the dorsal surface of the distal end.

In dorsal view, metacarpal IV is as long as metacarpal III but slenderer, and the cross section of the shaft is sub-circular. The proximal surface is also flat with sub-elliptical outline, but the ventrolateral margin is more concave than in metacarpals II and III. The distal articulation surface is asymmetric, the medial condyle larger than the lateral one. However, the lateral condyle expands and tapers ventrolaterally, which in distal view makes the outline sub-trapezoidal. No collateral ligament pits are present, there is only a slight depression midway on the dorsal part of the distal surface (no clear fossa running all the way dorsoventrally), and the dorsal extensor ligament pit is very shallow. The proximal outline of metacarpal V is also sub-elliptical, but with a convex surface.

The phalanges of each digit become progressively smaller and decrease in length : width ratio, except in digit IV where phalanx 4 is longer than phalanx 3 (which is the only phalanx as short as it is wide). All non-terminal phalanges are similar in shape, being dorsoventrally flattened and transversely expanded at both ends with a more expanded proximal portion than the distal one. The proximal surfaces are gently concave dorsoventrally for articulation with the previous manual element (metacarpal or phalanx), and the outlines are sub-triangular with a straight ventral margin. Shafts are narrowest at mid-length or slightly distal to this point, and sub-elliptical in cross section. The distal articulations possess two ventrally expanded condyles that are fairly symmetrical, presenting a trapezoidal outline that is ventrally concave in distal view. Collateral ligament pits are present on both medial and lateral condyles, as well as an extensor ligament pit on the dorsal surface of the distal end.

The ungual of digit I is mediolaterally compressed and slightly dorsoventrally flattened, not strongly curved along its length, and is slightly mediolaterally asymmetrical, more expanded on the medial surface than laterally. A small ungual is tentatively identified as belonging to digit IV, based on size, articulation with the fourth phalanx of digit IV, and the typical archosaur condition of bearing five phalanges in the fourth digit [67]. Preservation is poor, but the ungual is dorsoventrally flattened, slightly lateromedially curved in dorsal view, the dorsal surface is smoother than the ventral side, and the ventral surface shows little curvature. A medial neurovascular groove is visible.

The phalangeal formula is known only for digits I and IV, each possessing two and five phalanges, respectively. The distal-most preserved phalanges of digits II and III possess well-developed distal articular surfaces indicating that at least one more phalanx would be present in each digit, whereas the number of elements in digit V is unknown. Suchian taxa with preserved manual elements, such as multiple specimens of *Postosuchus* spp. [67,68] and *Ticinosuchus ferox* [69], as well as the aetosaur *Stagonolepis* spp. [70,71], indicate that a possible manual phalangeal formula for *Mambawakale ruhuhu* is 2-3?-4?-5-3?.

### 3.6. Phylogenetic relationships

**Figure 15.**
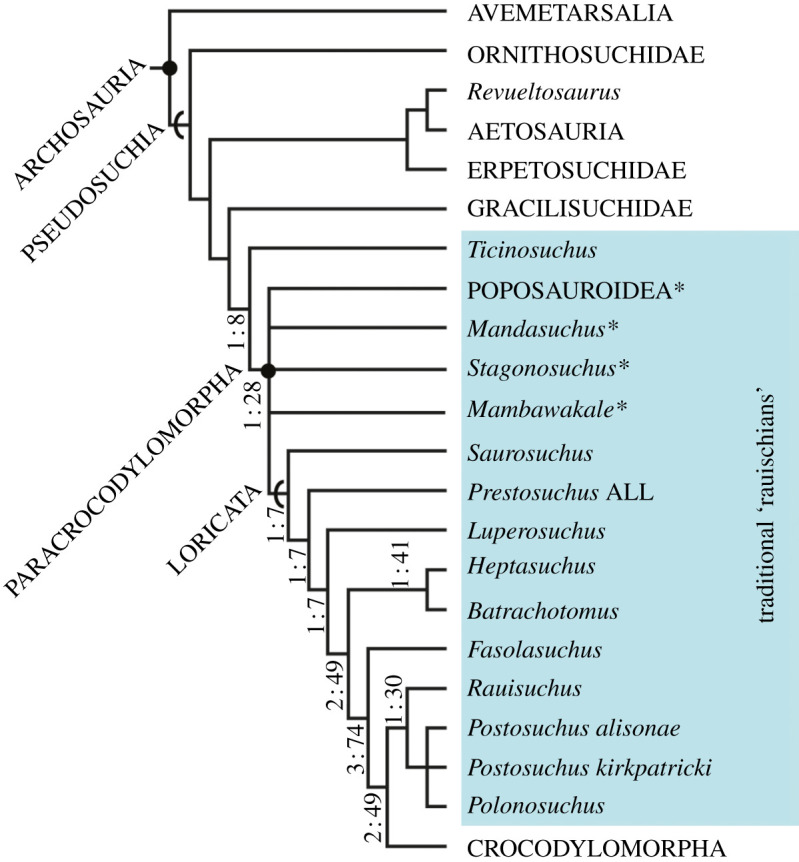
Results of the primary phylogenetic analysis of early archosaur relationships conducted here including *Mambawakale ruhuhu. Mambawakale ruhuhu* was found in a polytomy with two other Manda Beds taxa (*Mandasuchus* and *Stagonosuchus*) within Paracrocodylomorpha. Abbreviated strict consensus tree derived from 288 most parsimonious trees (MPTs) with a length of 1540 steps (consistency index = 0.332; retention index = 0.750) (see electronic supplementary material, figure S1 for full results). Bremer supports (left) and bootstrap (right) are provided for the ‘rauisuchian’ taxa (=Paracrocodylomorpha and *Ticinosuchus ferox*). Asterisks highlight paracrocodylomorph taxa from the Manda Beds.

Our main phylogenetic analysis recovered 288 most parsimonious trees (MPTs) with lengths of 1540 steps (consistency index = 0.332; retention index = 0.750). In the strict consensus (figure 15; electronic supplementary material, figure S1) *Mambawakale ruhuhu* is positioned at the base of Paracrocodylomorpha in a polytomy with two other taxa from the Manda Beds, *Mandasuchus tanyauchen* and *Stagonosuchus nyassicus*, as well as Loricata (with *Saurosuchus galilei* as the sister to all other loricatans) and Poposauroidea (with *Qianosuchus mixtus* as the sister to all other poposauroids). In the MPTs, the three genera from the Manda Beds (*Mambawakale, Mandasuchus* and *Stagonosuchus*) are variously recovered subtending basally within either Loricata or Poposauroidea.

Our second analysis, with the inclusion of *Nundasuchus songeaensis,* resulted in a similar position of *Mambawakale ruhuhu* (288 MPTs with lengths of 1554 steps; consistency index = 0.329; retention index = 0.748; electronic supplementary material, figure S2) at the base of Paracrocodylomorpha in a polytomy with two other taxa from the Manda Beds, *Mandasuchus tanyauchen* and *Stagonosuchus nyassicus*, as well as Loricata (with *Saurosuchus galilei* as the sister to all other loricatans) and Poposauroidea (with *Qianosuchus mixtus* as the sister to all other poposauroids). *Nundasuchus songeaensis* falls outside the clade containing *Ticinosuchus ferox* and Paracrocodylomorpha.

Overall, the phylogenetic position of *Mambawakale ruhuhu* is not compellingly resolved, being poorly supported and constrained (figure 15; electronic supplementary material, figure S1) in both analyses, but a few details about its relationships are clear. Most of the phylogenetic signal derives from the maxilla and the premaxilla and their associated dentition, and these elements of *M. ruhuhu* possess many archosaur synapomorphies, including palatal processes of the maxillae that meet on the midline and an antorbital fossa present on the dorsal portion of the posterior process of the maxilla (see [6] and [37] for discussion of characters). Within Archosauria, clear pseudosuchian character states are more difficult to identify and the character states placing *M. ruhuhu* within the clade have a broad distribution and are mostly clearly homoplastic. For example, the pterygoid of *Mambawakale* lacks dentition, similar to the condition in many pseudosuchian groups, but the loss of pterygoid dentition is complex and probably occurred multiple times within Archosauriformes (e.g. [14]).

It is not surprising that the most parsimonious phylogenetic position of *Mambawakale ruhuhu* is at the base of Paracrocodylomorpha given the combination of character states in the facial region of the skull. However, the known material of *M. ruhuhu* possesses very few character states that are not plesiomorphic or highly homoplastic within Paracrocodylomorpha. The seemingly autapomorphic posterodorsal (=maxillary) process of the premaxilla is ventrally low posteriorly, like that of poposauroids, but also extends posteriorly like that of other loricatans. Moreover, it appears that the maxilla of *M. ruhuhu* participates in the external naris, which is a character state found in many poposauroids (e.g. *Xilousuchus sapingensis*) but also within Loricata (e.g. *Batrachotomus kupferzellensis*; [32]). The maxilla of *M. ruhuhu* is dorsoventrally tall like that of *Saurosuchus* and *Prestosuchus,* and in contrast with that of poposauroids. Furthermore, the lateral surface of the maxilla has a shallow antorbital fossa that lacks a distinct bounding ridge, similar to the condition in the early diverging loricatans *Saurosuchus* and *Prestosuchus*. Postcranially, the known material of *M. ruhuhu* possesses only a few characters that can be scored. Unfortunately, several of the skeleton regions preserved for this taxon either do not carry much phylogenetic signal in this part of the archosaur tree (cervical vertebrae) or are poorly sampled throughout the tree with unclear character-state distributions (manus, palate).

In sum, the relationships of *Mambawakale ruhuhu* are poorly resolved, but our best current estimate of its phylogenetic position is within Pseudosuchia, near the base of Paracrocodylomorpha. Although imprecise and not strongly supported, this phylogenetic position is important to understanding the taxonomic diversity of archosaurs in the Manda Beds and the general diversification of pseudosuchians in the Middle to early Late Triassic.

## 4. Discussion

*Mambawakale ruhuhu* is the largest archosaur known from cranial remains from the Manda Beds and is among the larger-headed archosaurs from the Middle to Late Triassic. Several other pseudosuchian archosaurs are already known from the Manda Beds that potentially overlap the estimated size of *M. ruhuhu* and have often been named on the basis of fragmentary material. Here, we draw comparisons between *M. ruhuhu* and these other Manda Beds taxa, as well as with smaller taxa from the Manda Beds that theoretically potentially represent younger individuals of *M. ruhuhu*.

*Stagonosuchus nyassicus* [8] (recombined as *Prestosuchus nyassicus* by [36]) is a large-bodied suchian known from two fragmentary skeletons representing mostly postcranial material [36,72,73]. Only very limited cranial material (a right articular and one element identified as either a postorbital or a jugal) is referred to *S. nyassicus*, and does not facilitate comparisons with *Mambawakale ruhuhu*, in which the postorbital is unknown and the articular poorly preserved, although the latter appears less robust in *M. ruhuhu* than in *S. nyassicus* [72]. The only postcranial elements which are known in both *S. nyassicus* and *M. ruhuhu* are the atlantal intercentrum and axis. Although poorly preserved in available material of both species, the morphology of the axial centrum is similar, being anteroposteriorly compressed such that the length of the centrum is slightly shorter than its dorsoventral height. The available specimens of both taxa also appear to represent individuals of similar sizes. It is possible therefore that *S. nyassicus* and *M. ruhuhu* could represent the same species, but this cannot be determined based upon available material.

*Parringtonia gracilis* [9] is an erpetosuchid pseudosuchian known from a fragmentary holotype [18] and three referred specimens that represent nearly the entire skeleton [16]. The cranial anatomy of the monotypic *P. gracilis* is clearly highly distinct from that of *Mambawakale ruhuhu*. In particular, the external naris is proportionately much smaller in *P. gracilis*, the premaxillary dentition is not strongly heterodont, and the maxilla of *P. gracilis* is characterized by a low tooth count (5) with teeth restricted to the anterior portion of the bone and a more extensively developed and deeply recessed antorbital fossa [16,18].

*Hypselorhachis mirabilis* is a ctenosauriscid poposauroid known from a single dorsal vertebra collected at locality U11/2, approximately 10 km west of the type locality of *Mambawakale ruhuhu* (see [10,48]). The vertebra is relatively large and is characterized by a very elongate neural spine (5.5 times centrum height). No other material has been referred to this taxon to date. The lack of overlapping material makes comparisons between *H. mirabilis* and *M. ruhuhu* difficult although both represent larger-bodied taxa than the majority of archosaur remains within the Manda Beds collections. Notably, ctenosauriscids typically have cervical vertebrae (including the axis and cervical 3) with anteroposteriorly elongated centra [5,50], whereas the anterior cervical vertebrae of *M. ruhuhu* are anteroposteriorly compressed, inconsistent with a ctenosauriscid identification.

*Nundasuchus songeaensis* [14] is a pseudosuchian known from a single partial skeleton much smaller than the only known specimen of *Mambawakale ruhuhu*. There is limited overlap between the known material of the monotypic *N. songeaensis* and *M. ruhuhu*, but the two appear distinct. Notably, the pterygoid of *N. songeaensis* possesses three rows of pterygoid teeth on its ventral surface and the dentary lacks the elongate symphysis present in *M. ruhuhu* and is distinguished by anteroposteriorly extending grooves on its anterolateral surface that are absent in the latter [14]. *Mambawakale ruhuhu* also lacks the strongly laterally projecting surangular ridge present in *N. songeaensis*.

*Mandasuchus tanyauchen* [11] is a paracrocodylomorph known from five specimens representing much of the postcranial skeleton but very little of the skull [11]. All known material is much smaller in size than that of *Mambawakale ruhuhu*. The limited cranial material (two maxillae) of *Mandasuchus tanyauchen* is poorly preserved, but the maxilla of *Mambawakale ruhuhu* appears to be proportionately deeper and to have a relatively longer portion of the bone anterior to the ascending process and an ascending process that is not strongly compressed from anterolateral to posteromedial (the strongly compressed ascending process was recognized as an autapomorphy of *Mandasuchus tanyauchen* by [11]). Moreover, the centra of the axis and cervical 3 of *Mandasuchus tanyauchen* are anteroposteriorly elongated [11], and clearly distinct from the anteroposteriorly short centra of the anterior cervicals of *Mambawakale ruhuhu*. Although changes in cranial morphology and vertebral proportions through ontogeny are poorly understood in early pseudosuchians, it seems likely that *Mandasuchus tanyauchen* and *Mambawakale ruhuhu* are distinct taxa.

*Mambawakale ruhuhu* can therefore be distinguished with confidence from *Parringtonia* and *Nundasuchus*, and probably also from *Hypselorhachis* and *Mandasuchus*. *Mambawakale ruhuhu* and *Stagonosuchus* cannot be confidently distinguished from one another based on available material, but overlapping material is extremely limited and there is no positive evidence to support identification of them as the same species. For this reason, we consider *Mambawakale ruhuhu* to be distinct.

**Figure 16.**
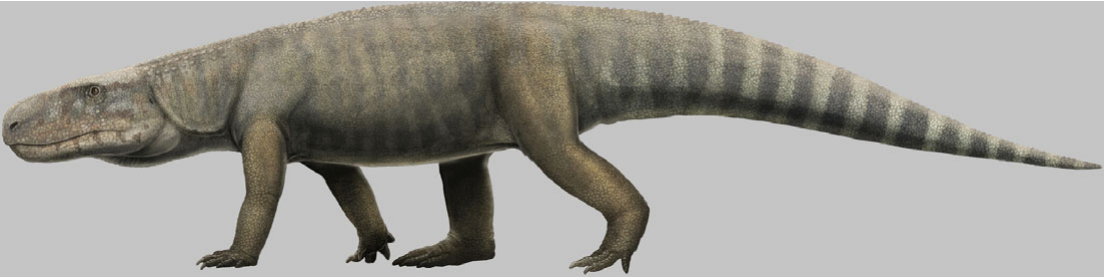
Life reconstruction of *Mambawakale ruhuhu* by Gabriel Ugueto, who retains the copyright. Only the skull, mandible and a few postcranial elements are known for *Mambawakale ruhuhu*, so the rest of the body, tail and limbs are reconstructed based on the anatomy of hypothesized close relatives of similar size.

Regardless of its precise affinities, the type specimen of *Mambawakale ruhuhu* is one of the largest pseudosuchian specimens recovered from the Manda Beds and was probably an apex predator within the ecosystem. Comparison to *Prestosuchus chiniquensis* from the late Middle Triassic of Brazil [54], one of the largest and best known pseudosuchians from this time interval, suggests a complete skull length for *M. ruhuhu* in excess of 75 cm. This would make it one of the largest pseudosuchians known from the Middle Triassic (figure 16), and comparable in size to or slightly larger than *Etjosuchus recurvidens* from the Middle Triassic of Namibia [61]. This large size is combined with a unique morphology including an unusual and much enlarged external naris. *Mambawakale ruhuhu* was the last of the unpublished ‘Charig taxa’ from the Manda Beds [26], now finally published nearly sixty years after first discovery and mention in the literature. It adds to the exceptional diversity of the Manda Beds archosaur assemblage, with at least nine species now known (*Asilisaurus kongwe*, *Hypselorhachis mirabilis*, *Mambawakale ruhuhu*, *Mandasuchus tanyauchen*, *Nundasuchus songeaensis*, *Nyasasaurus parringtoni*, *Parringtonia gracilis*, *Stagonosuchus nyassicus*, *Teleocrater rhadinus*), providing a rich insight into the complexity of the ecosystems occupied by early archosaurs.

## Data accessibility

Data are provided as electronic supplementary material [74] or have been uploaded to the NHM Data Portal or MorphoSource (links in the text).
